# Effects of Habitat Partitioning on the Distribution of Bacterioplankton in Deep Lakes

**DOI:** 10.3389/fmicb.2019.02257

**Published:** 2019-10-04

**Authors:** Nico Salmaso

**Affiliations:** Research and Innovation Centre, Fondazione Edmund Mach, San Michele all’Adige, Italy

**Keywords:** deep lakes, meromixis, bacterioplankton, biodiversity, habitat partitioning, amplicon sequence variants, high throughput sequencing

## Abstract

In deep lakes, many investigations highlighted the existence of exclusive groups of bacteria adapted to deep oxygenated and hypoxic and anoxic hypolimnia. Nevertheless, the extent of bacterial strain diversity has been much less scrutinized. This aspect is essential for an unbiased estimation of genetic variation, biodiversity, and population structure, which are essential for studying important research questions such as biogeographical patterns, temporal and spatial variability and the environmental factors affecting this variability. This study investigated the bacterioplankton community in the epilimnetic layers and in the oxygenated and hypoxic/anoxic hypolimnia of five large and deep lakes located at the southern border of the Alps using high throughput sequencing (HTS) analyses (16S rDNA) and identification of amplicon sequence variants (ASVs) resolving reads differing by as little as one nucleotide. The study sites, which included two oligomictic (Garda and Como) and three meromictic lakes (Iseo, Lugano, and Idro) with maximum depths spanning from 124 to 410 m, were chosen among large lakes to represent an oxic-hypoxic gradient. The analyses showed the existence of several unique ASVs in the three layers of the five lakes. In the case of cyanobacteria, this confirmed previous analyses made at the level of strains or based on oligotyping methods. As expected, the communities in the hypoxic/anoxic monimolimnia showed a strong differentiation from the oxygenated layer, with the exclusive presence in single lakes of several unique ASVs. In the meromictic lakes, results supported the hypothesis that the formation of isolated monimolimnia sustained the development of highly diversified bacterial communities through ecological selection, leading to the establishment of distinctive biodiversity zones. The genera identified in these layers are well-known to activate a wide range of redox reactions at low O_2_ conditions. As inferred from 16S rDNA data, the highly diversified and coupled processes sustained by the monimolimnetic microbiota are essential ecosystem services that enhance mineralization of organic matter and formation of reduced compounds, and also abatement of undesirable greenhouse gasses.

## Introduction

In deep lakes, seasonal differences in vertical water density gradients driven by water temperature and salinity are a key feature in the control of stratification dynamics and patterns (Wetzel, 2001; Kallf, 2002). In holomictic lakes, the cooling of surface waters in the coldest months is sufficient to trigger the complete circulation and vertical homogenization of the water column every year. Within this typology, oligomictic lakes mix only irregularly. Conversely, in meromictic lakes, circulation in the winter months affects only the upper portion (mixolimnion) of the water column, whereas the deeper stratum of water (monimolimnion) is perennially separated from the surface by a steep salinity gradient (the chemolimnion) (Wetzel, 2001). As a consequence of complete isolation and oxidation of sinking particulate organic matter, in meromictic lakes the monimolimnion is almost (<1–2 mg L^–1^) or completely depleted of oxygen, causing low redox potential conditions. The monimolimnion is often rich in phosphorus and reduced nitrogen compounds. Conversely, while reduced substances accumulate in the monimolimnion, biota in the surface illuminated layers and dark mixolimnion may be deprived of essential nutrients (Humayoun et al., 2003).

Depending on the lake physiography, illumination and nutrients, different communities develop in the epilimnion of lakes, including well-diversified photosynthetic cyanobacteria populations (Newton et al., 2011; Salmaso et al., 2018a). In the epilimnion and hypolimnion of holomictic lakes, and in the oxygenated mixolimnetic layers of meromictic lakes, the metabolic activities of microorganisms can rely on a sufficient replenishment of oxygen. In depleted-O_2_ layers, microorganisms have to switch electron acceptors from oxygen to other compounds such as nitrate, manganese, iron, sulfate, and carbon dioxide (Stumm and Morgan, 1996; Humayoun et al., 2003). In the metalimnion of relatively shallower meromictic lakes, oxygen diffusing down from the surface and sulfide diffusing up from the hypolimnion, provide a niche for photosynthetic and non-photosynthetic sulfur-oxidizing bacteria (Storelli et al., 2013; Bush et al., 2017). As a result, several studies based on classical culture-independent approaches showed the occurrence of specific bacterioplankton populations exclusively occurring in the oxygenated (Urbach et al., 2001; Pollet et al., 2011; Okazaki and Nakano, 2016) and hypoxic/anoxic hypolimnia (De Wever et al., 2008; Casamayor et al., 2012; Mori et al., 2013; Kubo et al., 2014). A number of investigations, based on the application of high throughput sequencing (HTS) approaches, identified a high operational taxonomic unit (OTU) richness in the hypolimnetic layers shaped by oxygen availability (Garcia et al., 2013; Llirós et al., 2014; Kurilkina et al., 2016). Nevertheless, insights into the richness and spatial extension/distribution of bacteria in large and deep lakes remain largely to be explored (Rinke et al., 2013).

The lakes Garda, Maggiore, Como, Iseo, Lugano, and Idro are part of the group of deep lakes located at the southern border of the Alps [deep southern perialpine lakes (DSL) (Ambrosetti and Barbanti, 1992)]. Owing to their different maximum depths, deep mixing dynamics and oxygenation patterns, these lakes are excellent sites to study the selection of bacterial populations along oxic-hypoxic and light gradients. In a selection of these lakes, including both oligomictic and meromictic typologies, the application of CARD-FISH showed more pronounced differences in the vertical profile of prokaryoplankton than those observed between spring and summer (Hernández-Avilés et al., 2018). These changes were interpreted as caused by the physical and chemical differences between the epilimnetic and deep hypolimnetic layers. However, it was not possible to test the extent of these effects on the whole community biodiversity and the potential ecological selection of taxa adapted to specific lakes and layers.

Thanks to the vertical partition of the water layers, ecological selection in deep lakes has the potential to lead to the development of communities composed of different populations adapted to different local habitats. In this perspective, the work aims to clarify the extent and pattern in the bacterial community diversification and how this diversification may be explained by environmental variations. A specific objective of this work is to characterize the bacterioplankton community composition (BCC) in the upper and oxygenated layers, dark oxygenated layers, and deep hypoxic layers of DSL, quantifying the influence of light and oxygen concentrations (as a proxy of redox conditions) on the potential selection of specific bacterial lineages. A specific emphasis will be given to Cyanobacteria. This group of bacteria is widely represented with several adaptations in many aquatic and terrestrial ecosystems (Jungblut et al., 2010; Whitton, 2012; Jasser et al., 2013). Moreover, many species belonging to this phylum are able to develop huge blooms (Reynolds and Walsby, 1975; Bullerjahn and Post, 2014; Davis et al., 2019), producing a wide variety of toxic compounds that can strongly deteriorate the quality of water resources used for drinking and bathing purposes (Meriluoto et al., 2017a, b). Cyanobacteria are an issue also for the DSL where they are represented by several toxigenic species producing either hepatotoxins (microcystins; *Planktothrix rubescens* and *Microcystis aeruginosa*) or neurotoxins (anatoxins; *Tychonema bourrellyi*) (Cerasino et al., 2017).

## Materials and Methods

### Study Sites

**FIGURE 1:**
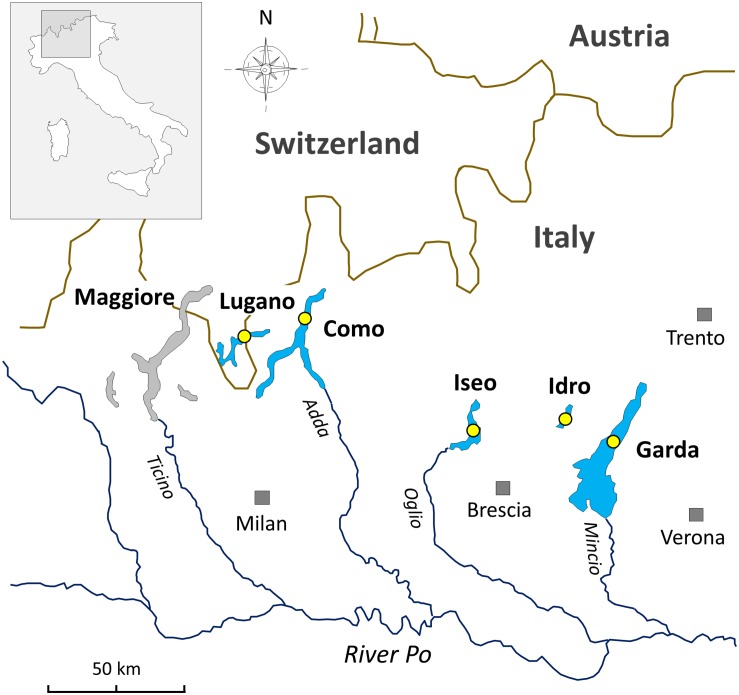
Geographical location of the deep southern perialpine lakes (DSL) investigated in this work. The yellow circles indicate the sampling points.

The lakes included in this work are located at the southern border of the Alps (Figure 1). The status of investigations in this selection of lakes and in the other large and deep lakes surrounding the Alpine chain has been reviewed by Salmaso et al. (2018b, and references therein). In this regard, in the Alpine region, an operational demarcation of large and deep lakes was defined by surface (S), maximum depth (z_*m*_), and volume (V) values set at S 10 km^2^, z_*m*_ 50 m, and V 0.5 km^3^. The two largest lakes included in this work, i.e., Garda and Como, are oligomictic and oligo-mesotrophic; they have surface and maximum depths of 368 and 146 km^2^, and 350 and 410 m, respectively. The two last complete vertical circulation events were between 2004 (Garda) and 2005–2006 (Garda and Como) (Rogora et al., 2018). Since then, hypolimnetic oxygen concentrations were above 7 mg L^–1^. Lakes Iseo, Lugano, and Idro are meromictic and characterized by a trophic state between mesotrophy and meso-eutrophy; water surface and maximum depths are 62, 28 and 11 km^2^, and 251, 288, and 124 m, respectively (Ambrosetti and Barbanti, 1992). In lakes Iseo and Lugano a larger overturn with hypolimnetic (<150 m) O_2_ concentrations increasing up to 8 and 2.5 mg L^–1^, respectively, was observed in 2006 and, partially, 2005 (Rogora et al., 2018); hypoxia and anoxia conditions restored immediately after 2–4 years. In Lake Idro, low oxygenation from 60 to 100 m and anoxia from 100 m to the bottom were documented since 1969 (Viaroli et al., 2018). Meromixis in lakes Iseo, Lugano, and Idro was triggered by a number of related factors, including the smaller water volumes compared to the other oligomictic lakes, geographical location sheltered from winds (Lugano, Idro), water warming (Lepori and Roberts, 2015; Pareeth et al., 2017), eutrophication and enhanced calcite precipitation, and supply of dissolved minerals from groundwater springs (Idro) (Rogora et al., 2018; Salmaso et al., 2018b; Viaroli et al., 2018).

### Sampling and Laboratory Measurements

**FIGURE 2:**
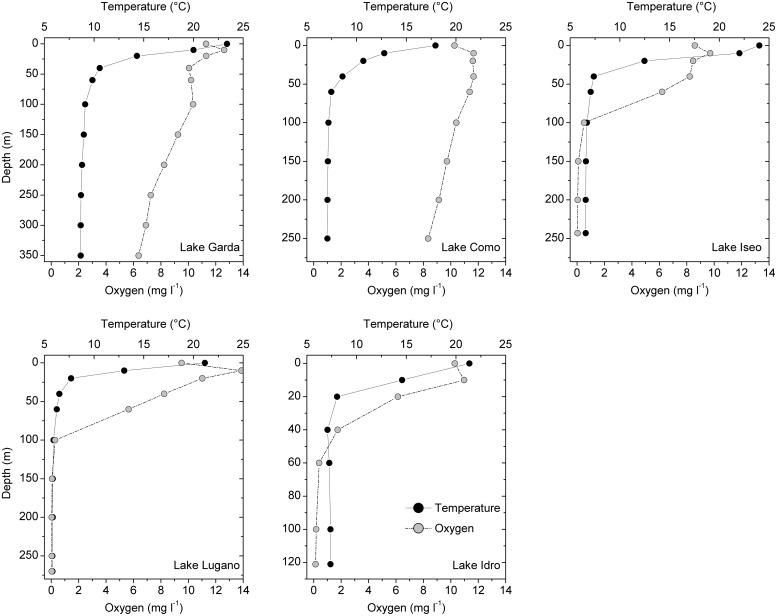
Vertical measurements of water temperatures and oxygen concentrations in the two oligomictic (Garda and Como) and three meromictic (Iseo, Lugano, and Idro) lakes at discrete depths. Vertical scales are identical in lakes Como, Iseo, and Lugano. Samples for the determination of other chemical compounds and for HTS analyses were collected at the same depths.

Samplings and measurements were carried out in 2016 during the stratification period in the deepest zones of the lakes Garda (5 July), Iseo (29 August), Lugano (northern basin, 21 June) and Idro (23 August); in Lake Como (14 June), samplings were carried out at the station of Dervio, in the northern basin (z_*max*_ = 270 m). The sampling stations in lakes Garda, Como, and Iseo are part of the Italian and European Long Term Ecological Research network (LTER^1^ ^,2^). Constant volumes of water were collected in each layer with a 5 L Niskin bottle for successive subsampling. Vertical profiles of temperature and oxygen were carried out using underwater probes (Idronaut, Seacat-Seabird, WTW probes). In the upper layers, samples were collected at 0, 10, 20, 40, and 60 m. Additional samples were collected, every 50 m or less, in the hypolimnetic layers (see Figure 2). The overall number of samples collected in the five lakes was 11 (Garda), 9 (Como), 9 (Iseo), 10 (Lugano), and 7 (Idro). Conductivity (at 20°C), pH, nitrogen compounds (NO_3_-N, NH_4_-N), total phosphorus (TP), soluble reactive phosphorus (SRP), reactive silica (Si), alkalinity and major ions (Ca, Mg, Na, K, Cl, and sulfate) were determined in a single laboratory (FEM) using standard analytical procedures (APHA-AWWA-WEF, 2005; Cerasino and Salmaso, 2012).

### DNA Extraction and Amplification

For HTS analyses, aliquots of water were filtered through 25 mm polycarbonate Isopore membranes (Merck) with 0.2 μm pore size. Filtered volumes ranged between around 500 and 1000 mL, depending on the quantity of suspended particles, and until near-clogging of the filters. Filters were stored at −20°C until DNA extraction with MO BIO PowerWater^®^ DNA Isolation Kit (MO BIO Laboratories, Qiagen, United States). DNA concentrations, measured with a NanoDrop ND-8000 (Thermo Fisher Scientific, Inc., Waltham, MA, United States), ranged between 5 and 46 ng μL^–1^. PCR amplification of bacterial sequences was carried out by targeting a ∼ 460-bp fragment of the 16S rRNA gene variable regions V3–V4 using the specific bacterial primer set 341F (5′-CCTACGGGNGGCWGCAG-3′) and 805Rmod (5′-GACTACNVGGGTWTCTAATCC-3′) with overhang Illumina adapters. The final barcoded library was sequenced on an Illumina^®^ MiSeq (PE300) platform. A detailed description of procedures is reported in Salmaso et al. (2018a). Sequences in FASTQ format were deposited to the European Nucleotide Archive (ENA) with study Accession No. PRJEB33405.

### Bioinformatic Pipelines and Downstream Analyses

Sequences were analyzed using the DADA2 package 1.10 (Callahan et al., 2016a) in R 3.5.1 (R Core Team, 2018) and Bioconductor 3.8 (Huber et al., 2015). The DADA2 algorithm is more sensitive and more specific than common bioinformatic pipelines based on the OTU identification, and resolves amplicon sequence variants (ASVs) that differ by as little as one nucleotide (Callahan et al., 2017). The results are exact ASVs that replace the traditional OTUs obtained by pipelines that cluster reads using subjective fixed global clustering threshold (generally 97%) or local thresholds (Swarm method) (Nguyen et al., 2016). Taxonomic assignment was carried out using the RDP naive Bayesian classifier method described in Wang et al. (2007) and the SILVA v. 132 ribosomal reference database (Quast et al., 2013; Glöckner et al., 2017) with a 80% minimum bootstrap confidence threshold. The corresponding general tree was built after alignment of sequences and phylogenetic analysis carried out using the R packages DECIPHER 2.10 and phangorn 2.4.0 (Callahan et al., 2016b), respectively. The workflow and intermediate steps are illustrated in the Supplementary Methods. After the application of the DADA2 pipeline, a total of 3514 ASVs were obtained, with an average sequence length of 417 bp. The number of reads per sample ranged between 13448 and 48922 (mean ± SD, 30809 ± 8790).

The ASVs abundance table, taxonomy, DNA reads and environmental data were imported into the R package phyloseq 1.28.0 (McMurdie and Holmes, 2013). Chloroplasts, mitochondria, unclassified/non-bacterial sequences were removed from the original dataset, obtaining 3298 ASVs. After inspection of the distribution of sample sequencing depth, the ASVs table was rarefied without replacement to 13367 sequences per sample, obtaining a final table with 3216 ASVs. Alpha diversity (observed ASVs, Chao1 index, and Shannon diversity) and beta-diversity (Bray and Curtis) were computed following Salmaso et al. (2018a). Differences in alpha diversity between samples and layers were estimated using the Kruskal–Wallis rank sum test (KW). Ordination of samples was carried out by non-metric multidimensional scaling (NMDS) computed on a Bray and Curtis (BC) dissimilarity matrix, and vector fitting procedures (Salmaso et al., 2018a). Differences in bacterial composition between groups of samples were tested using PERMANOVA computed on the same BC distance matrix used in NMDS, with 9999 bootstraps and function adonis in R vegan 2.5.5 package (Oksanen et al., 2018).

Correlations between environmental variables and the bacterial community in the 5 lakes and selected layers were calculated by computing Mantel tests (Legendre and Legendre, 1998; Oksanen et al., 2018). The environmental distance matrix was computed using a set of standardized environmental variables (temperature, pH, conductivity, O_2_, SRP, NO_3_-N, NH_4_-N, Si, Alk, SO_4_). A second distance matrix was computed including only a subset of variables linked to stratification (water temperature) and major chemical gradients (pH, O_2_, NH_4_-N). The bacterial dissimilarity matrix was computed using the same methods used in NMDS. The significance of the statistic was evaluated by 9999 permutations of rows and columns of the dissimilarity matrix.

The differential distribution of taxa along the vertical oxygen gradient was tested using DESeq2 1.22.1 package in R (Love et al., 2014). Computations were carried out on the original (non-rarefied and un-normalized) abundance ASV table. Additionally, for every *k* species (or higher level taxonomy), the observed optimum environmental levels of oxygen were estimated by computing the average values – weighted by the corresponding abundances values – of the O_2_ concentrations in the corresponding samples where the *k* species were identified. Species optima (u_*k*_), and species tolerances (t_*k*_), were computed using standard approaches (ter Braak and van Dame, 1989) (R script in Supplementary Code).

The distribution of ASVs in the selected phylum Cyanobacteria was evaluated by mapping abundances on a phylogenetic tree built, after aligning sequences with MAFFT 7.427 (Katoh and Standley, 2013), using phyML 3.1 (Guindon et al., 2010) and the R package phyloseq. Potentially poorly aligned positions and divergent regions of the alignment were checked using Gblocks (Talavera and Castresana, 2007). The DNA substitution model (GTR + I + G) was selected after calling PhyML 3.1 with the phymltest function in the R package ape (Paradis, 2012; Salmaso et al., 2016). Only taxa identified at least at the genus level were included in the analysis. The outgroup was chosen from non-photosynthetic Cyanobacteria (NCY, Melainabacteria, unclassified taxa belonging to the order Caenarcaniphilales; Soo et al., 2014), after previous verification of their position in the general phylogenetic tree.

## Results

### Vertical Physical and Chemical Gradients

The five lakes showed steep surface temperature gradients up to 40–50 m (Garda, Como, Iseo) and 20 m (Lugano, Idro) (Figure 2). Dissolved oxygen concentrations in the two oligomictic lakes (Garda and Como) showed only a minor vertical decrease, ranging between 6 and 13 mg L^–1^. In the meromictic lakes, O_2_ concentrations between 5.7 and 6.2 mg L^–1^ were measured up to the depths of 60 m (Iseo and Lugano) and 20 m (Idro). Below 60 m, O_2_ concentrations in these three lakes were always below 0.5 mg L^–1^. Based on the stratification patterns and O_2_ concentrations, three layers were identified. In the five lakes, the upper oxygenated and productive layer included sampling depths between the surface and 20 m (“epi_oxy”; 15 samples); the dark oxygenated layers included all the hypolimnetic sampling depths in the oligomictic lakes (Garda and Como), and the depths at 40 and 60 m in lakes Iseo and Lugano (“hyp_oxy”; 18 samples); the third layer included the deep, dark and hypoxic sampling depths in the three meromictic lakes, below 100 m (Iseo and Lugano) and 40 m (Idro) (“hyp_hypox”; 13 samples).

The vertical gradients of oxygen were paralleled by similar changes in the vertical distribution of nitrate and ammonium nitrogen (Supplementary Figure 1). In lakes Garda and Como, NO_3_-N begun to increase after the first 20–30 m, up to around 320 μg L^–1^ (Garda) and 750 μg L^–1^ (Como) in the deep layers; conversely, NH_4_-N was generally below 20 μg L^–1^. Apart from the increase of nitrate up to 20–50 m, both NO_3_-N and NH_4_-N in the three meromictic lakes showed opposite vertical patterns (Supplementary Figure 1). In lakes Garda, Como and Iseo, TP concentrations at the surface (0–20 m) were between 7 and 9 μg L^–1^, whereas in lakes Lugano and Idro values were 20 and 12 μg L^–1^, respectively. In the deep layers (>20 m), TP reached the highest concentrations in the meromictic lakes (averages between 88 and 204 μg L^–1^). All the five lakes showed vertical decreasing values of pH and vertical increasing values of conductivity, Si, Alk, Ca, and Mg. In particular, the hypolimnion of the meromictic lakes showed low-medium pH (range: 7.4–8.3), high conductivity values (229–454 μS cm^–1^), and high concentrations of calcium (37–71 mg L^–1^) and alkalinity (117–209 mg L^–1^) and, especially in Lake Idro, sulfate (7.6–86 mg L^–1^), magnesium (7.2–20 mg L^–1^), and silica (0.8–5.4 mg L^–1^). Corresponding ranges in lakes Garda and Como were: pH, 7.4–8.2; conductivity, 169–240 μS cm^–1^; calcium, 25–39 mg L^–1^; alkalinity, 71–134 mg L^–1^; sulfate, 11–27 mg L^–1^; magnesium, 5.6–8.5 mg L^−1^; silica, 0.3–2.3 mg L^–1^. Differences in the distribution of Cl, Na, and K (i.e., conservative ions, or ions not susceptible of chemical precipitation) in the oligomictic and meromictic lakes were less apparent.

### Alpha Diversity

**FIGURE 3:**
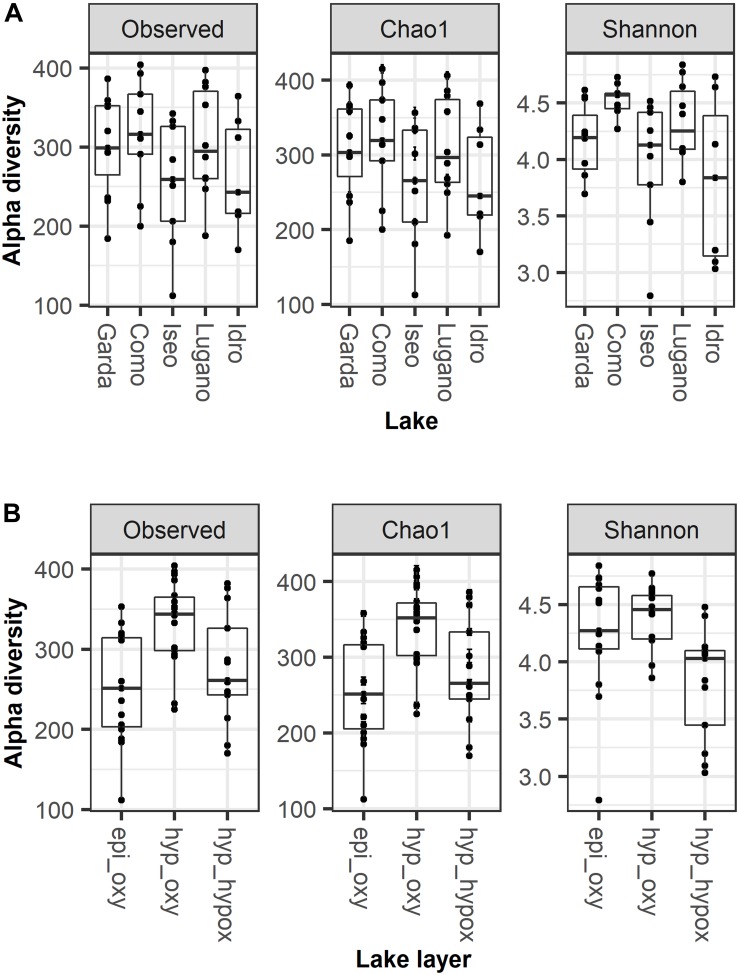
Alpha diversity estimates in **(A)** the 5 lakes and **(B)** the three main water layers; epi_oxy, oxygenated upper layers (0–20 m); hyp_oxy, oxygenated hypolimnion; hyp_hypox, hypoxic/anoxic hypolimnion; the three layers are defined as in the text. Boxes report the median and hinges as 25th and 75th quartiles.

The number of ASVs in the 5 lakes was generally comparable (Figure 3A; KW, *P* = 0.32). The median number of ASVs per lake was between 243 (Idro) and 316 (Como). Chao1 values closely followed the observed number of ASVs. Compared to Lake Como, the Shannon diversity in the other lakes showed a larger dispersion and lower values (KW, *P* = 0.04). The number of ASVs was higher in the oxygenated hypolimnion (median 344) than in the other two layers (251–261) (Figure 3B; KW, *P* < 0.01). Shannon diversity was higher in the oxygenated layers (Figure 3B; KW, *P* < 0.01).

### Distribution of Bacterioplankton

**FIGURE 4:**
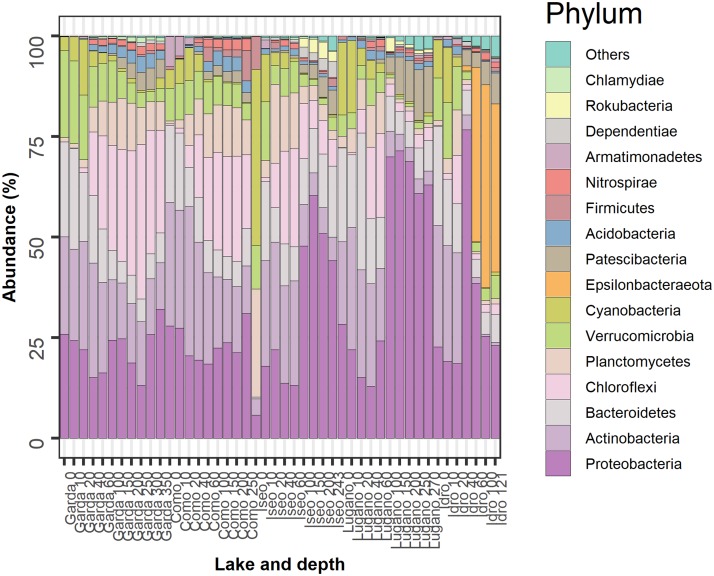
Temporal development of the 16 more abundant phyla in the 5 lakes. Samples are coded by the name of the lake and depth (meters). The bars report the percentage contributions on the sample totals. The bacterial groups were selected identifying, for each lake, the 12 more abundant phyla. The high fraction of cyanobacteria at the surface of Lake Iseo was caused by a higher development of *Dolichospermum lemmermannii*.

The most abundant bacterioplankton phyla were Proteobacteria, Actinobacteria, Bacteroidetes, Chloroflexi, Planctomycetes, Verrucomicrobia, Cyanobacteria, and Epsilonbacteraeota. The relative contributions of these groups along with those of eight other phyla contributing with a minor fraction of reads are reported in Figure 4. In the two oligomictic lakes Garda and Como, differences in the vertical distribution of the main phyla were less apparent compared to the three meromictic lakes, and mostly limited to particular groups such as the Chloroflexi (family Anaerolineaceae) and Actinobacteria (*hgcI clade*, *CL500-29 marine_group*). Other dominant taxa in the two oligomictic lakes included Proteobacteria (*Ralstonia*, *Limnohabitans*), Bacteroidetes (*Fluviicola*, *Flavobacterium*), and Planctomycetes (*CL500-3*), whereas photosynthetic Cyanobacteria were mostly represented by *Tychonema bourrellyi*. In lakes Garda and Como, the dominant orders almost coincided (10 out of 12; Supplementary Figure 2).

The three meromictic lakes showed a strong increase of Proteobacteria in the upper and/or deep hypolimnion. Lake Idro had a peculiar presence of Epsilonbacteraeota (formerly Epsilonproteobacteria, Proteobacteria) at higher depths. Differences in the bacterial composition in the hypoxic layers were already apparent at the order level (Supplementary Figure 2). In Lake Iseo, the surface sample showed a lower abundance of Proteobacteria and Actinobacteria, and a greater fraction of Cyanobacteria (*Dolichospermum lemmermannii*) and Planctomycetes (family Gemmataceae) (Figure 4). In the trophogenic layers, a further important contribution of Oxyphotobacteria (*Pseudanabaena*) was found in Lake Lugano.

The number of ASVs exclusively present in the single lakes overshadowed those common in two or more lakes (Supplementary Figure 3A). Differences were even more amplified considering the distribution in the three main lake layers (Supplementary Figure 3C); the hyp_hypox layer included the larger fraction of exclusive ASVs. Differences between layers were apparent also considering the two oligomictic lakes (Supplementary Figure 3E). Nevertheless, most of the differences were due to the occurrence of rarest ASVs, as shown in Supplementary Figures 3B,D,F, which included only the more common species selected after filtering out rarest ASVs that did not appear more than 10 times in at least two samples (995 ASVs).

### Relationship With Environmental Variables

**FIGURE 5:**
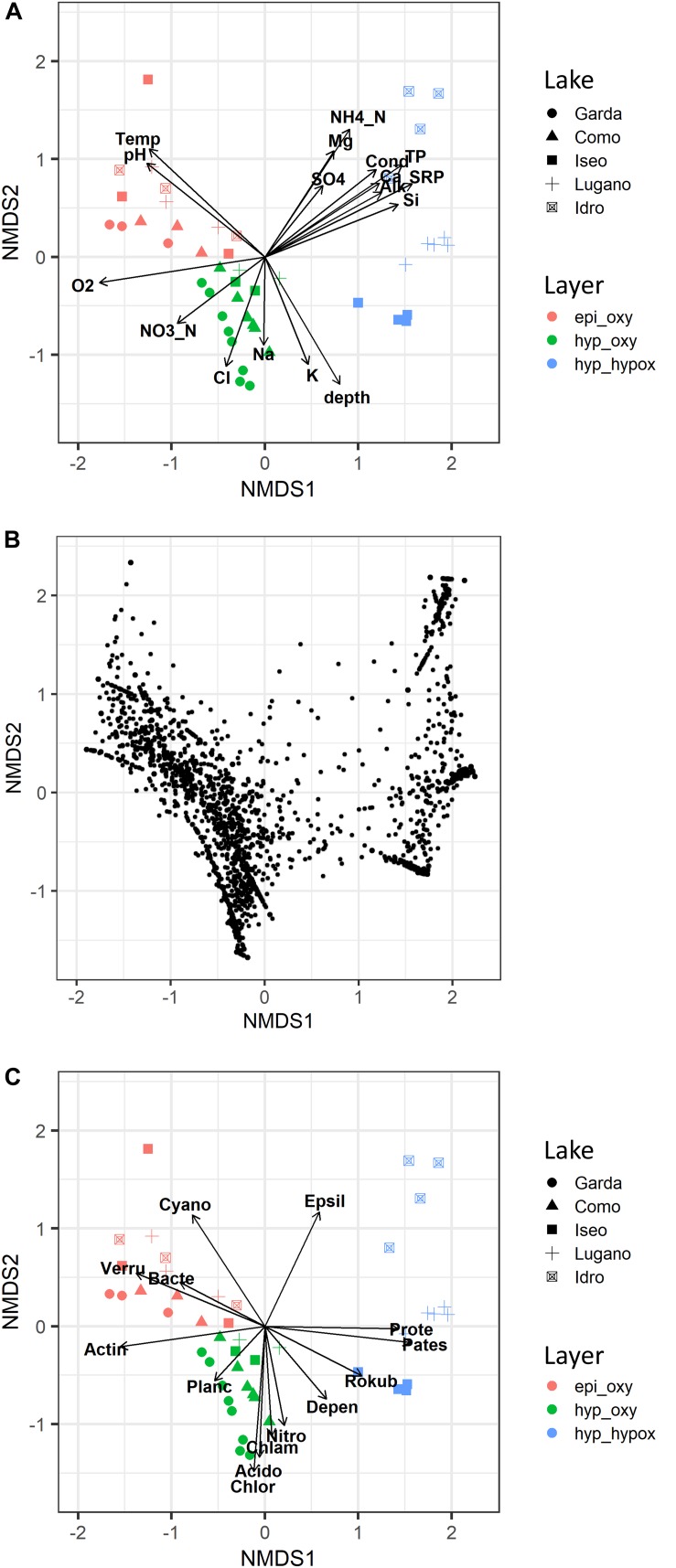
Non-metric multidimensional scaling (NMDS) ordination (stress = 0.09) of samples based on the bacterioplankton composition; samples are coded by lake name (symbols) and layer (colors). **(A)** Vector fitting of significant (*P* < 0.01) environmental variables: Temp, water temperature; Cond, water conductivity; O_2_, dissolved oxygen; SRP, soluble reactive phosphorus; TP, total phosphorus; NO_3__N, nitrate nitrogen; NH_4__N, ammoniacal nitrogen; Si, reactive silica; Alk, alkalinity; SO_4_, sulfate; depth, sampling depth; other major ions are indicated by the element name. **(B)** Ordination of ASVs. **(C)** Vector fitting of significant (*P* < 0.01) dominant phyla: Acido, Acidobacteria; Actin, Actinobacteria; Bacte, Bacteroidetes; Chlam, Chlamydiae; Chlor, Chloroflexi; Cyano, Cyanobacteria; Depen, Dependentiae; Epsil, Epsilonbacteraeota; Nitro, Nitrospirae; Pates, Patescibacteria; Planc, Planctomycetes; Prote, Proteobacteria; Rokub, Rokubacteria; Verru, Verrucomicrobia. epi_oxy, 0–20 m layer; hyp_oxy, oxygenated hypolimnion; hyp_hypox, hypoxic/anoxic hypolimnion.

Differences in the bacterial community in the 5 lakes and 3 main water layers were well-exemplified in the configurations obtained by NMDS (final stress, 0.09) (Figure 5A). Samples collected in the oxygenated upper layers, in the deep oxygenated layers, and in the deep hypoxic layers were grouped into three different zones (PERMANOVA, *P* < 0.001). A closer inspection of sites in the three layers allowed to identify differences in the positioning of samples belonging to the different lakes (PERMANOVA, *P* < 0.001), due to, e.g., the position of the samples of Lake Garda in the left area of the configuration, and to the three meromictic lakes, which showed a clear separation of the respective samples in the deep hypoxic hypolimnion (Figure 5A). The separation of samples was followed by a different positioning of ASVs (Figure 5B), which, in the case of the deep hypoxic layers, showed a clear grouping near the three meromictic lakes, suggesting the existence of peculiar deep communities. This was supported by the greater ASVs average dissimilarities between the samples of the deep hypoxic layers across lakes compared to the epi_oxy and hyp_oxy layers (Supplementary Figure 4).

Besides O_2_, samples in the upper oxygenated layers were characterized by higher temperature values and pH, whereas the deep oxygenated layers had higher concentrations of NO_3_-N, Cl, Na, and K (Figure 5A). Especially in Lake Idro, the deep hypoxic layers showed higher conductivity values, and higher concentrations of NH_4_-N, TP, SRP, Si, sulfate, alkalinity, Ca, and Mg. The vector fitting analysis carried out including the dominant phyla listed in Figure 4, confirmed the close association between different bacterial groups and specific water layers (Figure 5C), such as, among others, Proteobacteria and Epsilonbacteraeota in the deep hypoxic layers, and Cyanobacteria in the upper illuminated layers.

The correlations between the environmental variables and the community structure were highly significant considering both the pooled data (5 lakes and layers; Table 1A), and the single lakes (Table 1B) and layers (Table 1C) separately. In general, though characterized by lower *r* values, all the correlations based on the group of 4 variables were equally significant (epi_oxy, *P* < 0.05) or highly significant (lakes, and the layers hyp_oxy and hyp_hypox; *P* < 0.001).

**TABLE 1:** Association of the bacterial community structure with environmental factors based on **(A)** all the lakes and layers, **(B)** single lakes, and **(C)** single layers.

**A**			

**Lake/layers**	**Factor**	**Mantel *r***	***P***
ALL	10 var	0.72	<0.001
ALL	4 var	0.75	<0.001

**B**			

**Lake**	**Factor**	**Mantel *r***	***P***

Garda	10 var	0.93	<0.001
Como	10 var	0.89	<0.001
Iseo	10 var	0.78	<0.001
Lugano	10 var	0.89	<0.001
Idro	10 var	0.91	<0.001
Garda	4 var	0.86	<0.001
Como	4 var	0.86	<0.001
Iseo	4 var	0.71	<0.001
Lugano	4 var	0.82	<0.001
Idro	4 var	0.87	<0.001

**C**			

**Layer**	**Factor**	**Mantel *r***	***P***

epi_oxy	10 var	0.41	<0.05
hyp_oxy	10 var	0.58	<0.001
hyp_hypox	10 var	0.73	<0.001
epi_oxy	4 var	0.33	<0.05
hyp_oxy	4 var	0.50	<0.001
hyp_hypox	4 var	0.58	<0.001

*The analyses were carried out by applying Mantel tests using Bray and Curtis dissimilarity matrices for ASVs abundances, and Euclidean distance matrices for groups of standardized environmental variables. Significance was assessed with 9999 permutations. The four variables include temperature, O_2_, pH and NH_4_-N; the 10 variables include, additionally, conductivity, SRP, NO_3_-N, Si, alkalinity and SO_4_.*

### Characterization of the Dominant Genera Along the Oxygenation Gradient

Differential prevalence of taxa in one of the three reference water layers (epi_oxy, hyp_oxy, and hyp_hypox) was evaluated testing their abundances in one selected layer compared to the remaining layers (Table 2 and Supplementary Table 1). The analysis has been carried out at the level of genus, including results significant at least at *P* < 0.05, and with a log2 fold change (Love et al., 2014) > 4. As expected, all the genera in the epi_oxy layer were aerobic and adapted to metabolic functions requiring O_2_ (u_*k*_ optima ranges of O_2_ between 8.7 and 11.9 mg L^–1^; Supplementary Table 1), and/or potentially associated with human and animal hosts (Table 2A). Besides *Dolichospermum*, the most abundant genus in this group was *Flavobacterium*, which was present with a sizeable relative number of reads also in the oxygenated hypolimnion. A number of families with u_*k*_-O_2_ ranging between 9.0 and 11.2 mg L^–1^ tested positive for their prevalence in the epilimnetic layers (Table 2A). As expected, genera characteristics of the hyp_oxy layer (Table 2B) mostly inhabited the two oligomictic lakes Garda and Como, with a very limited presence in the three meromictic lakes. The O_2_ optima range for this group of taxa was 7.1–9.7 mg L^–1^.

**TABLE 2:** Differential distribution of genera along the vertical oxygen gradient.

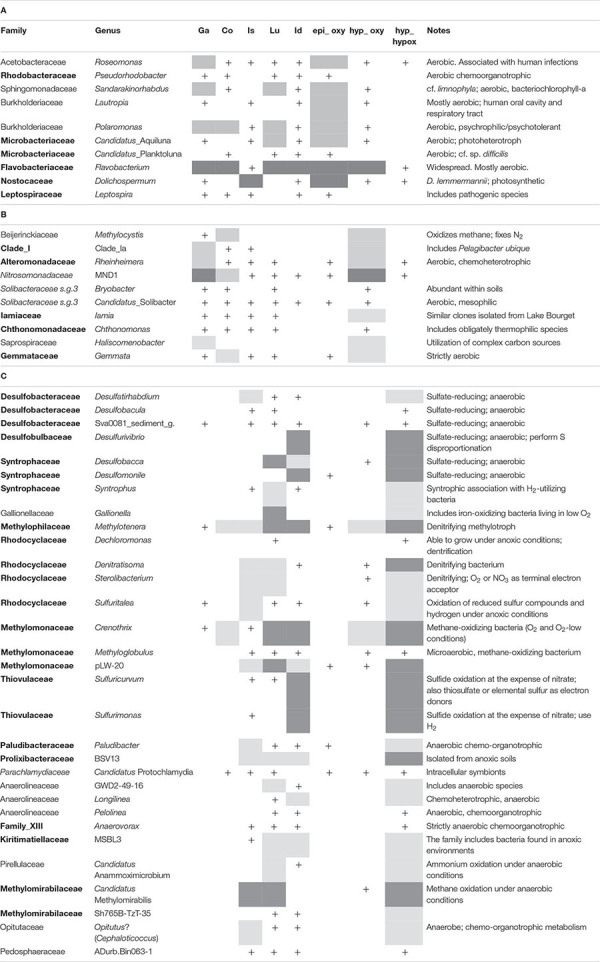

*Taxa recorded with higher abundances (DESeq2, *P* < 0.05) in **(A)** the epilimnetic and **(B)** the hypolimnetic oxygenated layers, and **(C)** the hypoxic and anoxic monimolimnia. Cumulative relative abundances are highlighted as: +, 0% < reads < 1%; light gray, 1% ≤ reads < 5%; dark gray, reads ≥ 5%. For each layer in **(A–C)**, families with abundances significantly different from the other two layers were reported in bold (*P* < 0.01) and italic (*P* < 0.05). Ga, Garda; Co, Como; Is, Iseo; Lu, Lugano; Id, Idro. epi_oxy, oxygenated upper layers (0–20 m); hyp_oxy, oxygenated hypolimnion; hyp_hypox, hypoxic/anoxic hypolimnion. Family and genus names are based on the SILVA 132 taxonomy. The complete taxonomic table and additional information are reported in Supplementary Table 1. The bacteria listed in the table are known to present a wide array of adaptations and metabolic functions; only a few of the most characteristics are reported under the column “Notes.”*

The deep and hypoxic layers showed a number of classifiable genera typically present in the three meromictic lakes (Table 2C). Consistently with the distribution of ASVs at the phylum level (Figure 4), many genera belonged to the Proteobacteria, mostly Delta- and Betaproteobacteria, and Epsilonbacteraeota, as well as to other less represented phyla. u_*k*_-O_2_ ranged between 0.05 and 1.4 mg L^–1^. The taxa identified belonged to genera that are known to be strictly or facultative anaerobic, or microaerobic, and linked to a wide variety of redox reactions at low O_2_ conditions. Most genera belonging to the class Deltaproteobacteria were associated with sulfate reduction reactions, with *Desulfomonile* and *Desulfurivibrio* in lakes Idro and Lugano, *Desulfobacca* in both lakes, and *Desulfatirhabdium* in the three meromictic lakes. Along with other members of the Desulfobulbaceae, *Desulfurivibrio* is known also to perform disproportionation of elemental sulfur to sulfide and sulfate (Poser et al., 2013). A variety of oxidation processes under anaerobic and/or microaerobic conditions was carried out by a wide array of bacterial groups. Processes included methane oxidation (families Methylomonaceae, Methylomirabilaceae) and ammonium oxidation (*Candidatus* Anammoximicrobium); besides part of the family Rhodocyclaceae, denitrification can be performed by a wide array of microbial groups (including methanotrophs and methylotrophs). Other processes connected with known phenotypic characters included H_2_-utilization in syntrophic association (*Syntrophus*) and, particularly in Lake Idro, sulfide (and hydrogen) oxidation at the expense of nitrate (*Sulfuricurvum* and *Sulfurimonas*). *Sulfurimonas* have been shown not only to use sulfide as electron donors but also for example thiosulfate or elemental sulfur. In Lake Lugano, *Gallionella* included iron-oxidizing bacteria requiring at least a certain amount of O_2_. Further genera belonging to the less abundant phyla were linked to the mineralization of a wide variety of organic matter, including complex carbohydrates, cellulose, proteinaceous C, and organic acids (Table 2). Though it is difficult to clearly associate prevalent chemical functions to specific genera of bacteria (most of them are associated to multiple chemical transformations), the above descriptions provide an indication about the specialization of biologically mediated chemical processes in oxygen depleted hypolimnetic waters. Compared to the oxygenated layers, a few families emerged as typical monimolimnetic groups. Excluding the Methylophilaceae (u_*k*_-O_2_, 4.5) the optimum range of O_2_ of “monimolimnetic” families was between 0.06 and 1.45 mg L^–1^.

### Variability of ASVs

The reads identified at the genus level were characterized by a very variable number of ASVs, due to variability in how many ASVs were nested within each genus. In a number of genera, such as *Flavobacterium*, the variability in ASVs was very high (61) and, considering the high number of species potentially included in this genus, not straightforward to interpret (mean sequence similarity in the 61 ASVs was 94%). For this genus, it was actually possible to assign a species name to only a low fraction of ASVs, i.e., *F. pectinovorum*, *F. succinicans*, *F. paronense*, *F. chungnamense/koreense*, and *F. terrigena*, each one present with only one sequence variant. Most of the different variants were mostly present in 2 or 3 lakes, only rarely 5 (figure not shown).

**FIGURE 6:**
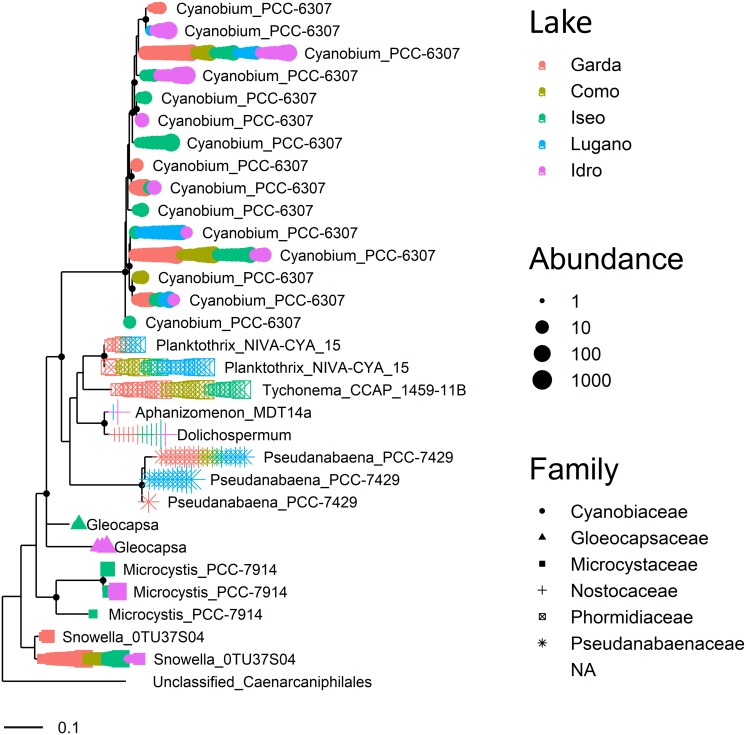
Maximum likelihood (ML) rooted topology of cyanobacteria identified at least at the genus level in the 5 lakes based on alignment of 16S rRNA gene fragments; the tree is rooted by an outgroup member of non-photosynthetic cyanobacteria (unclassified Caenarcaniphilales, Melainabacteria). Each symbol on the tip of the tree corresponds to a single sample; different symbols and colors correspond to the cyanobacterial families (with the exclusion of the outgroup, “NA”) and to the 5 lakes, respectively; the size of symbols is scaled according to abundance. The small black filled circles at the nodes indicate corresponding branch support aLRT-SH-like (Anisimova and Gascuel, 2006) values > 0.85.

A similar distribution characterized other genera, such as those included in Cyanobacteria (Figure 6). The largest group in this phylum was represented by 15 ASVs classified within the picocyanobacteria (0.8–1.4 μm), namely *Cyanobium* PCC-6307. The similarity between each pair of aligned sequences assigned to this taxon ranged between 94.8 and 99.8%. Of these, only one ASV was present in the 5 lakes, whereas the majority (8) was found in one or several samples in one unique lake (Figure 6). The distribution of the *Cyanobium* strain present in the five lakes showed significant differences (KW, *P* < 0.001), with a number of reads greater in Lake Garda. Conversely, the second more abundant strain present in four lakes did not show apparent differences (KW, *P* < 0.1, excluding Lake Lugano). At the species level, the two *Planktothrix* NIVA CYA 15 were assigned to the complex *P. agardhii/rubescens*. These two ASVs were distinguished by two sequence variants (AG). After a BLAST analysis, the more abundant ASV, (A), which was identified in all the lakes with the exclusion of Lake Idro, showed 100% similarity with many *P. rubescens/P. agardhii* sequences, including those previously associated to *P. rubescens* in the largest lakes south of the Alps (Salmaso et al., 2016). The distribution of this variant showed significant differences among lakes (KW, *P* < 0.001), with higher abundances in Lake Lugano. The second variant (G; lakes Garda, Iseo, and Lugano) was previously identified in Lake Garda using HTS approaches (Salmaso et al., 2018a). The sequence of the most abundant member of Nostocaceae (*Aphanizomenon* NIES 81), fully coincided (BLAST, 100%) with that of *Dolichospermum lemmermannii* (lakes Garda, Iseo, and Idro); the identification was confirmed also by previous observations carried out in all the DSL using a polyphasic approach (e.g., Capelli et al., 2017), and by further classification of reads using EzBioCloud (Yoon et al., 2017) and SILVA 132. The less abundant taxon among Nostocaceae coincided (99%) with *Aphanizomenon flos-aquae* (lakes Lugano and Idro). *Tychonema* sequences were identified, with one unique abundant oligotype, in lakes Como, Garda, and Iseo.

In this work, the new established group of non-photosynthetic Cyanobacteria was identified only in the three meromictic lakes, both in the oxygenated and hypoxic layers (Class Melainabacteria, orders Caenarcaniphilales, Gastranaerophilales, and Vampirovibrionales) and in the hypoxic layers (Class Sericytochromatia). The identity of the ASV attributed to Sericytochromatia was confirmed using a further downstream classification in SILVA 132, and evaluation of its position in the general phylogenetic tree. Considering the overall means, the relative abundances of NCY on the total of Cyanobacteria was however low, with maximum contributions < 6% in Melainabacteria and < 1% in Sericytocromatia.

The most impressive differences in the ASVs composition characterized the populations inhabiting the deep hypoxic/anoxic layers. Focusing on the most abundant taxa of Table 2C, all the genera were represented by at least one ASV exclusively present in one lake (Table 3). The high proportion of bases in common between each pair of sequences suggested that most of these genera, which were unclassified at the species level, were composed of the same or very closely related species. In several cases, only a very few ASVs were shared between two or more than one lake. It is worth to highlight the presence of unique ASVs of *Methylotenera* and *Crenothrix*, not shared with the meromictic lakes, in the two oligomictic lakes Garda and Como.

**TABLE 3:** Number of ASVs in the genera identified with higher abundances in the hypoxic and anoxic hypolimnia (Table 2C); only the most abundant genera (cumulative abundances > 1%) and with more than one ASVs have been included.

**Genus**	**ASVs**	**% sim**	**Iseo**	**Lugano**	**Idro**	**More lakes**
*Desulfatirhabdium*	3-3	99.1		2		1, Is_Lu_Id
*Desulfurivibrio*	3-3	99.4			3	
*Desulfobacca*	17-17	97.2^(a)^		9	8	
*Desulfomonile*	7-7	96.8			7	
*Syntrophus*	8-8	95.4		6	1	1, Is_Lu
*Methylotenera*	7-7	96.7		4		1, Is_Lu_Id – 1, lightgrayCo_Lu_Id – lightgray1, Ga_Co
*Sterolibacterium*	9-9	95.4	3	5		1, Lu_Is
*Sulfuritalea*	10-10	97.7	5	2		1, Is_Lu_Id – 1, Is_Id – lightgray1, Ga
*Crenothrix*	8-8	94.4	1	1		3, Lu_Id – 2, Is_Id – lightgray1, Ga_Co
pLW-20	7-7	98.6		3		4, Is_Lu_Id
*Sulfuricurvum*	4-4	96.2		1	2	1, Is_Id
*Sulfurimonas*	3-3	99.3			2	1, Is_Id
*Paludibacter*	14-13	97.4	5		4	4, Is_Id - 1, Is_Lu
BSV13	17-16	94.6	7	3	4	2, Is_Lu_Id - 1, Is_Id
GWD2-49-16	2-2	98.5		1		1, Lu_Id
*Longilinea*	2-2	92.1			1	1, Lu_Id
MSBL3	11-10	97.2^(b)^	1	5	3	1, Lu_Id - 1, Is_Id
*C.* Methylomirabilis	3-3	97.5	2			1, Is_Lu
*Opitutus*? (*Cephaloticoccus*)	2-2	98.1				1, Is_Lu_Id - 1, Is_Lu

*“ASVs” reports the number of sequence variants identified on the original and rarefied tables, respectively. “% sim” reports the mean percentage DNA base similarity among ASVs; ^(a,b)^ excluding two and one, respectively, singular and very rare sequences in Lake Lugano. The number of exclusive ASVs in the lakes refers to determinations made on the original table; “More lakes” reports the number of ASVs shared between two or more lakes; oligomictic lakes are highlighted in light gray. Abbreviations as in Table 2.*

## Discussion

This work demonstrated the existence of a clear link between the vertical partition of water layers and the ecological selection of different bacterial populations and groups adapted to different local habitats. The BCC showed a large and significant divergence in the three main water layers that were identified on the basis of the oxygen concentrations and the main vertical physical and chemical gradients, namely the upper and oxygenated layers, dark oxygenated layers, and deep hypoxic layers. The differentiation was detected at different taxonomic levels and was linked to the ecological selection of different taxa adapted to perform a variety of metabolic functions constrained and driven by the availability of oxygen (as a proxy of redox conditions) and chemical compounds. Further, the higher sequence resolution to marker gene surveys provided by the oligotyping approach allowed to identify several exclusive ASVs present in single lakes and/or specific water layers and depths. After a brief examination of the elements that should be taken into account when analyzing ASVs, these aspects will be addressed in the next sections.

### Interpretation of Oligotyping Results

The interpretation of data based on the identification of 16S rDNA oligotypes has to take into account several potential limitations intrinsic in the restricted sensitivity of this marker to resolve ecological and evolutionary variation between closely related lineages (Berry et al., 2017). The elucidation of ASVs diversity has to take into account the multicopy nature and intragenomic variability of the 16S rRNA gene (Větrovský and Baldrian, 2013; Stoddard et al., 2015; Callahan et al., 2017). Nevertheless, polymorphic sites in intragenomic 16S rRNA genes are scarce and occur at a much lower frequency than between 16S rRNA genes of different species (Espejo and Plaza, 2018). ASVs cannot be confused with species or clones (Dijkshoorn et al., 2000), rather they represent different oligotypes (Eren et al., 2013) of the same or different species and clones. As highlighted by Berry et al. (2017), the hypothesis that 16S rDNA oligotypes represent ecotypes or species like groups is still largely untested. Further, interpretation of ecological differentiation (differential traits) based on 16S rDNA oligotypes requires great care because many bacterial functional traits are not phylogenetically conserved (Martiny et al., 2013). For example, the investigations carried out in Lake Erie and several Michigan inland lakes showed that *Microcystis* 16S rDNA oligotypes were not monophyletic and that they could not be used to infer toxicity (Berry et al., 2017).

At evolutionary time scales, the 16S rRNA gene has intrinsic limitations when the aim is to look at fine scale genetic differentiation because its molecular evolutionary rate is very slow (Ochman et al., 1999; Bahl et al., 2011; Dvořák et al., 2012; Segawa et al., 2018). Nevertheless, substitution rates in the 16S rRNA genes are highly variable across bacterial species, strongly depending on the life strategies and habitats. For example, rates of 16S rRNA gene evolution are higher in cultures or obligate pathogens and symbionts due to smaller population sizes and increased levels of genetic drift (Kuo and Ochman, 2009). However, these rates are still not within the ecological timescales of common limnological investigations, including the analyzed dataset. Therefore, from an evolutionary perspective, the 16S rRNA gene may not resolve more recent evolutionary diversification within a lineage (Berry et al., 2017).

### Environmental Drivers and BCC Distribution

The bacterial communities in the DSL showed a different distribution in the single lakes and water layers. A distinguishing element in the NMDS configuration was well-represented by the presence of clusters of ASVs in correspondence the hypoxic layers of the three meromictic lakes.

Differences in the bacterial community structure were significantly associated with the main environmental variables. Based on the Mantel tests, besides the whole group of physical and chemical variables, bacteria showed a strong link with a subgroup of variables particularly representatives of the main physical and chemical gradients that contribute to differentiate the deep oligomictic and meromictic DSL, i.e., besides thermal gradients, pH, O_2_, and NH_4_-N (Garibaldi et al., 1997; Mosello and Giussani, 1997; Viaroli et al., 2018). These results supported the view that significant ecological processes contributed to assemble different bacterial communities adapted to different ecological conditions. Compared to the oxygenated layers, the association between BCC and environmental variables was even more pronounced in the deep hypoxic layers of the three meromictic lakes, highlighting the existence of a strong environmental filtering and peculiar deep habitats. These results were fully consistent with the identification by DESeq2 of specific groups of taxa inhabiting the oxygenated layers (epi-oxy and hyp_oxy) in the five lakes and the hypoxic layers (hyp_hypox) in the three meromictic lakes (see next sections).

The identification of genera typical of the three water layers was fully consistent with the corresponding optima (u_*k*_) for oxygen, which, in the hypoxic/anoxic layers, and in the oxygenated layers, were between 0.05 and 1.4 mg L^–1^, and 7.1 and 11.9 mg L^–1^, respectively. These optima values allowed to quantify the O_2_ concentration requirements of distinct taxa developing in distinct strata. At the same time, these values can contribute to compare, on a quantitative basis, the oxygen niche of the same group of taxa in other aquatic systems.

### Diversity and Quantitative Importance and Ecophysiology of Representative Bacteria in the Three Lake Layers

The significance of the presence of 16S rDNA oligotypes in specific water layers (Table 2) was supported by a high number of ASVs characterized by cumulative relative abundances greater than 1 and 5%. The establishment of distinct bacterial genera in distinct layers allowed inferring a variety of metabolic processes. Although the 16S rRNA gene do not intrinsically provide evidence on microbial metabolism, this information has been widely used to identify metabolisms associated with related phenotyped strains (Bowman and Ducklow, 2015). Besides direct approaches (physiology and metabolomics), more detailed information on metabolic and functional profiles should rely on other sequencing techniques, such as shotgun metagenomic sequencing (Ortiz-Estrada et al., 2019).

Species localized at the surface were well-known to belong to groups living in oxygenated environments. Among these, Flavobacteria are known to include commensal and opportunistic pathogens (Crump et al., 2001). Though a few species of *Flavobacterium* may grow weakly under micro- and anaerobic conditions, many species are obligately aerobic and occurring in a variety of terrestrial and aquatic environments, and food (Betts, 2006). Other typical taxa at the surface included genera potentially associated with human and animal hosts (e.g., *Roseomonas* and *Lautropia*), as well as aerobic, anoxygenic (such as *Sandarakinorhabdus*; Gich and Overmann, 2006) and oxygenic (Cyanobacteria; Whitton, 2012) phototrophic bacteria. In the oxygenated hypolimnion, Anaerolineales (unclassified Anaerolineaceae) contributed for a great fraction to the bacterial community. A recent growing number of investigations confirmed the presence of this group (specifically the CL500-11 lineage) in the oxygenated hypolimnia of lakes (Urbach et al., 2007; Okazaki et al., 2013, 2018). CL500-11 was identified using CARD-FISH also in the hypolimnion of Lake Garda (Okazaki et al., 2018). In the oxygenated hypolimnion of Lake Michigan, Denef et al. (2016) showed an important role played by this taxon in nitrogen-rich DOM mineralization. Up to now, only a few species have been isolated and included in the class Anaerolineae, all of which showed anaerobic growth (Okazaki et al., 2013), as the three genera identified in the anoxic hypolimnion (Table 2C). Overall, the characteristics of this class and corresponding lower taxonomical levels will require a better characterization.

In the three meromictic lakes, the presence of peculiar species (Supplementary Figure 3) was particularly apparent in the hypoxic/anoxic hypolimnia. In the DSL, the unique assemblages in the deep layers were the result of the ecological selection of bacteria adapted to low redox conditions. The high relative abundance of sulfate reducing bacteria in Lake Idro can be linked to the replenishment of calcium sulfate from subsurface springs (Viaroli et al., 2018). The presence of several methane oxidizing bacteria in the hypoxic layers was indicative of an important methanotrophic activity. In freshwater lakes, methanotrophs are mostly part of aerobic Gamma- and Alpha-proteobacteria (such as *Methylocystis* in oxygenated hypolimnia; Table 2B). In the hypoxic/anoxic layers of lakes Iseo and Lugano, an important methanotroph was *Candidatus* Methylomirabilis. This genus includes species that are known to oxidize methane using a unique pathway of denitrification that tentatively produces N_2_ and O_2_ from nitric oxide (NO) (Graf et al., 2018). Selected species, such as *Ca.* M. limnetica were found to bloom in the anoxic layers of the deep stratified Lake Zug, suggesting a niche for NC10 bacteria in the methane and nitrogen cycle (Graf et al., 2018). A further important methanotroph able to develop with oxygen as well as under oxygen-deficient conditions in lakes Lugano and Idro was *Crenothrix*. Oswald et al. (2017) demonstrated that an important fraction of upward-diffusing methane in stratified freshwater lakes was oxidized by *Crenothrix polyspora*. Overall, methane-oxidizing bacteria are a major biological sink of CH_4_, protecting Earth against the effects of this strong greenhouse gas. Conversely, during mineralization of organic matter, the production of methane in anoxic conditions is due to anaerobic Archaea (not analyzed in this work). Methanogenesis represents the largest biogenic source of methane on Earth (Vanwonterghem et al., 2016). Though documented also in the upper well-oxygenated layers (Grossart et al., 2011; Bogard et al., 2014), methanogenesis is the cause of significant production and accumulations of CH_4_ in hypoxic and anoxic hypolimnia (Brock, 1985). *Candidatus* Anammoximicrobium, a bacterium capable of ammonium oxidation under anaerobic conditions in the presence of nitrite (Khramenkov et al., 2013) was detected in lakes Lugano and Idro. Nevertheless, high concentrations of ammonium in these two lakes could suggest that anammox activity is negligible (or at least at levels that do not consume the ammonium pool) or that nitrite, not measured, is limiting. Denitrification processes were exemplified by the presence of two denitrifying genera in lakes Iseo and Lugano: *Denitratisoma* and *Sterolibacterium*; these bacteria were shown to be able to use also O_2_ as terminal electron acceptor (Tarlera and Denner, 2003; Fahrbach et al., 2006). The phylum Epsilonbacteraeota included two among the most abundant monimolimnetic organisms, i.e., *Sulfurimonas* and *Sulfuricurvum*. Both oxidize sulfide at the expense of nitrate (Haaijer et al., 2012); their high abundance in Lake Idro can be coupled to high sulfate concentrations and reduction. Further, *Sulfurimonas* species can grow with a variety of electron donors and acceptors, including O_2_ as an electron acceptor for selected species (Han and Perner, 2015). Overall, the observation that anoxic hypolimnia harbor putative sulfur cycling groups not present in polymictic hypolimnia, which are more frequently oxygenated, was confirmed also in smaller lakes (z_*m*_ = 5–22 m) (Linz et al., 2017).

Besides large and deep lakes (Kurilkina et al., 2016; Salmaso et al., 2018a), the high diversity of deep layers documented in the DSL is also consistent with previous results obtained in a group of medium size (*z*_*m*_ < 30 m) stratified lakes, which showed a higher number of unique OTUs in the hypolimnia than epilimnia (Schmidt et al., 2016). A few of the most representative taxa identified in the DSL were common to the more abundant genera found recently in other two meromictic (Lake Pavin, z_*m*_ = 92 m) and dimictic (Lake Aydat, z_*m*_ = 12 m) perialpine lakes (Keshri et al., 2018). Besides the core microbiome typical of one or both lakes (*clade hgcI* and *CL500-29 marine group*, *Limnohabitans*), the most represented genera in the anoxic zone were *Syntrophus, Methylotenera, Gallionella, Sulfurimonas, Desulfurivibrio*, and *Sulfuritalea*. These genera were mostly present in the meromictic lakes included in this work, further confirming how predominant vertical environmental gradients can affect the environmental filtering of bacterial communities.

### Identification and Distribution of ASVs and Genera in Lakes and Water Layers

The identification of genera in Tables 2, 3 cannot be considered exhaustive because, in the analyzed dataset, only the 40 and <4% of taxa were classified at least at the genus and species level, respectively. Besides highlighting the limitations of techniques based on amplicon sequencing based on short reads (Poretsky et al., 2014; Ranjan et al., 2016), the high fraction of unclassified taxa at the lower taxonomic ranks contributed partly to confirm the existence of a fraction of diversity still not described in taxonomic databases (Menzel et al., 2015, 2016; Sunagawa et al., 2015). This incompleteness is mostly due to the limits of classical culture-based approaches in the estimation of environmental biodiversity (Lloyd et al., 2018; Overmann et al., 2019). Due to current fast technological developments, the detection of uncultured environmental “species” using metagenome-assembled genomes (MAGs) is expected to grow exponentially, calling for an update of present nomenclatural approaches (Oren and Garrity, 2018; Rosselló-Móra and Whitman, 2019). A paradigmatic example is provided by the recent discovery of non-photosynthetic cyanobacteria in a wide variety of environments (Di Rienzi et al., 2013; Soo et al., 2017) and in the perialpine lakes (Salmaso et al., 2018a; Monchamp et al., 2019). Excluding one isolated species (Soo et al., 2015), NCY are presently recognized and described from MAGs (Glöckner et al., 2017), without being formally described in the nomenclatural systems of prokaryotes (ICP) and algae fungi and plants (ICN) (Komárek et al., 2014; cf. Guiry and Guiry, 2019), and without clear described ecological roles and functions (but see Soo et al., 2015). On the other side, the taxonomic annotations in databases from predictions from sequences rather than authoritative assignments based on studies of type strains or isolates are not free of complications (Edgar, 2018).

A few classified genera included a high number of ASVs. At the genus level, though characterized by a high dispersion, the association between abundances and ASVs diversity was highly significant (Spearman ρ = 0.68, *P* < 0.001), highlighting the general relationship between sequencing depth and ASVs numbers. This was exemplified by the high number of ASVs in the genera identified with higher abundances in the hypoxic and anoxic hypolimnia (Table 3) and in the phylum Cyanobacteria. In this group, the higher number of ASVs was found in the smallest cell−size Cyanobacteria (*Cyanobium*), i.e., the fraction roughly corresponding to the picocyanobacteria (Jasser and Callieri, 2017). The high diversity of this functional fraction was recognized, using oligotyping methods (Eren et al., 2013), also in previous investigations carried out in Lake Garda (Salmaso et al., 2018a). In the case of Cyanobacteria investigated in previous studies, such as *Planktothrix rubescens* (D’Alelio et al., 2013; Salmaso et al., 2016), it was possible to confirm the existence of two sequence variants already identified in Lake Garda using oligotyping methods (Salmaso et al., 2018a). Conversely, filaments of *Tychonema bourrellyi* were identified with only one ASV, and only in the lakes where their presence was for the first time discovered using microscopical and phylogenetic methods (Shams et al., 2015; Salmaso et al., 2016). The appearance of this cryptogenic (*sensu* Kokociński et al., 2017) species was documented, for the first time in Lake Maggiore in 2004–2005. Since then, excluding lakes Lugano and Idro, *Tychonema* showed an increasing emergence in all the DSL. The discovery of *Tychonema* in the largest DSL was cause of concern, because of the ability of this species to produce neurotoxins (anatoxins; Shams et al., 2015; Cerasino et al., 2016). Analogously, the unique ASV detected in the populations of the bloom forming *Dolichospermum lemmermannii* (Figure 6) was consistent with the equivalence of 16S rDNA sequences in strains previously isolated in lakes Garda, Como, Iseo, and Lugano (Capelli et al., 2017).

While the presence of specialized bacterial communities in the monimolimnia of the three meromictic lakes was widely expected, the existence of groups of different ASVs was less obvious. Though not independent on the denoising strategy used (Nearing et al., 2018), the extent of ASVs differentiation was however apparent for several genera and species. A few dominant genera as well as ASVs were exclusively found in the deep layers of only one or two meromictic lakes. Considering the high 16S rDNA base similarity, most of the ASVs could represent strains belonging to the same species. The uneven distribution of taxa does not have a clear-cut explanation. Starting from the assumption that no barriers can prevent the migration of hypolimnetic inhabitants between the hypolimia of different lakes, the results of this study suggest that the exclusive (or dominant) presence of genera and species can be explained by ecological selection due to differences in environmental conditions in different lakes and strata.

The results obtained in this work have to be considered representative of the stratification period. As previously demonstrated, in large lakes BCC in the trophogenic layers can show strong temporal fluctuations following the temperate seasonal climate patterns (Salmaso et al., 2018a). Therefore, differences are expected to be mainly manifest in the layers mostly affected by climatic fluctuations and mixing dynamics, i.e., the surface layers, whereas the BCC in the more stable and not or only partially mixed deep hypolimnia should be less affected. Nevertheless, considering that at present the range of seasonal variation of BCC in the deepest layers is unknown, this speculation must be considered as a hypothesis to be verified on the basis of the investigations currently underway on a seasonal basis.

## Conclusion

The establishment of meromixis in deep lakes opens the way to the creation of new isolated, dark, hypoxic and anoxic habitats, drastically changing the biogeochemical gradients and processes along the water column. In the deep meromictic lakes south of the Alps, the formation of habitats completely segregated for long periods of time sustained the ecological selection and development of diversified bacterial communities. The highly diversified and coupled processes sustained by the monimolimnetic microbiota are essential ecosystem services that enhance mineralization of organic matter and formation of reduced compounds, and also abatement of undesirable greenhouse gasses. Though much less marked, the presence of distinctive populations was substantiated, confirming previous works (Denef et al., 2016; Okazaki et al., 2017, 2018), also in the oxygenated hypolimnia. Finally, though ASVs does not necessarily reflect phylogenetically consistent populations (Berry et al., 2017), this study confirms the utility of oligotyping based methods for distinguishing sequence types along ecological gradients. ASVs have an intrinsic biological meaning as a DNA sequence (Callahan et al., 2017). In perspective, their use will contribute providing a more solid basis to compare biodiversity in a wide spectrum of habitat types, including large lakes.

## Data Availability Statement

The datasets generated for this study can be found in the European Nucleotide Archive (ENA) with study accession PRJEB33405.

## Author Contributions

NS was involved in all aspects of the manuscript; see Acknowledgments for technical support.

## Conflict of Interest

The author declares that the research was conducted in the absence of any commercial or financial relationships that could be construed as a potential conflict of interest.

## Footnotes

1 http://www.lteritalia.it

2 http://www.lter-europe.net/
