# Supplementary material for: Effects of Habitat Partitioning on the Distribution of Bacterioplankton in Deep Lakes
Source: Front Microbiol. 2019 Oct 4;10:2257. doi: 10.3389/fmicb.2019.02257 (PMC6788347; doi:10.3389/fmicb.2019.02257)
Supplement: Supplementary file 1 [file Data_Sheet_1.PDF]

Supplementary methods

DADA2 pipeline.

The pipeline followed all the steps outlined in the tutorial of DADA2 version 1.10 (https://benjjneb.github.io/dada2/index.html) (Callahan et al., 2017). Computations were carried out on a machine equipped with 8 threads Xeon processor and 16 Gb RAM memory, using Linux Ubuntu 18.10, R 3.5.1 (R Core Team, 2018) and Bioconductor 3.8 (Huber et al., 2015).

After examination of quality profiles, forward (F) and reverse (R) reads were truncated at 275 bp and 220 bp, respectively, to remove low quality regions; truncLen parameters were large enough to maintain around 30 nucleotides (nts) of overlap for F and R sequences; at the same time, forward (17 nts) and reverse (21 nts) primers were trimmed; the maximum number of "expected errors" (EE) allowed in a read was set to 2; reads were truncated at the first instance of a quality score less than or equal to 4; finally, after truncation, sequences with indeterminate nucleotides (Ns) were discarded (DADA2 does not allow Ns). Filtering and trimming were performed with the function `filterAndTrim`, and parameters `truncLen=c(275,220), trimLeft = c(17, 21),maxN=0, maxEE=c(2,2), truncQ=4, rm.phix=TRUE, minLen = 50, compress=TRUE, multithread=TRUE, matchIDs=TRUE`).

The amplicon errors were modeled and corrected using the DADA2 algorithm (error model parameter learning) and default parameters. To reduce computation time, sequences were then dereplicated, combining all identical reads into "unique sequences". After inference of true amplicon sequence variants (ASVs) for each sample (Callahan et al., 2016a), forward and reverse ASVs were merged; paired reads that did not exactly overlap were removed. ASVs shorter than 401 or longer than 429 bp where removed. Chimeras were detected and removed from the obtained sequences.

Taxonomy was assigned to each of the ASVs using the naïve Bayesian classifier method (Wang et al., 2007) and the SILVA 132 reference database (Quast et al., 2013) using `assignTaxonomy`; the minimum bootstrap confidence for assigning a taxonomic level (`minBoot`) was increased from 50 (default) to 80 (more stringent). Species assignment was performed using the `addSpecies` function, which assigns Genus-species binomials to the input sequences based on exact matching against the reference fasta database, with `allowMultiple` option set to TRUE.

Callahan, B. J., McMurdie, P. J., and Holmes, S. P. (2017). Exact sequence variants should replace operational taxonomic units in marker-gene data analysis. *ISME J.* 11, 2639–2643. doi:10.1038/ismej.2017.119.

Callahan, B. J., McMurdie, P. J., Rosen, M. J., Han, A. W., Johnson, A. J. A., and Holmes, S. P. (2016a). DADA2: High-resolution sample inference from Illumina amplicon data. *Nat. Methods* 13, 581–583. doi:10.1038/nmeth.3869.

Callahan, B. J., Sankaran, K., Fukuyama, J. A., McMurdie, P. J., and Holmes, S. P. (2016b). Bioconductor Workflow for Microbiome Data Analysis: from raw reads to community analyses. *F1000Research* 5, 1492. doi:10.12688/f1000research.8986.2.

Huber, W., Carey, V. J., Gentleman, R., Anders, S., Carlson, M., Carvalho, B. S., et al. (2015). Orchestrating high-throughput genomic analysis with Bioconductor. *Nat. Methods* 12, 115–121. doi:10.1038/nmeth.3252.

Quast, C., Pruesse, E., Yilmaz, P., Gerken, J., Schweer, T., Yarza, P., et al. (2013). The SILVA ribosomal RNA gene database project: improved data processing and web-based tools. *Nucleic Acids Res.* 41, D590-6. doi:10.1093/nar/gks1219.

R Core Team (2018). *R: A language and environment for statistical computing*. Vienna: R Foundation for Statistical Computing.

Wang, Q., Garrity, G. M., Tiedje, J. M., and Cole, J. R. (2007). Naive Bayesian classifier for rapid assignment of rRNA sequences into the new bacterial taxonomy. *Appl. Environ. Microbiol.* 73, 5261–7. doi:10.1128/AEM.00062-07.
