# Supplementary material for: Effects of Habitat Partitioning on the Distribution of Bacterioplankton in Deep Lakes
Source: Front Microbiol. 2019 Oct 4;10:2257. doi: 10.3389/fmicb.2019.02257 (PMC6788347; doi:10.3389/fmicb.2019.02257)
Supplement: Supplementary file 2 [file Data_Sheet_2.pdf]

```
### Function waps (weighted averages using phyloseq-class objects)

## Nico Salmaso, 11 July 2019
## UPDATES: GitHub hts-tools/metatools

## For every i species, or higher taxonomic level, the observed optimum environmental levels of selected
variables are estimated by computing the averages – weighted by the abundances values – of the variable
values in the corresponding samples where the i species were identified. Species optima (uk), and
tolerances (tk), were computed using standard approaches (ter Braak and van Dame, 1989, and Salmaso, 2003).
# inputps, a phyloseq object
# varweight, the name of the numeric variable to analyze (e.g. "oxygen", "pH", "age", etc.)
# taxopt, the taxonomic level to be analyzed (e.g. "Genus", "Family" etc.)
# ranktax, if TRUE, the output table includes full taxonomic information
## example:
# reswaps <- waps(inputps = psobject, varweight = "Oxygen", taxopt = "Genus", ranktax = FALSE)

waps <- function (inputps = NULL, varweight = NULL, taxopt = NULL, ranktax = FALSE)
{
require(phyloseq)
require(data.table)
psg <- tax_glom(physeq=inputps, taxrank=taxopt, NArm=TRUE, bad_empty=c(NA, "", " ", "\t"))
psmor <- psmelt(psg)
psmordt <- as.data.table(psmor)
# remove rows with varweight = NA (and "Abundance" = NA)
psmdt <- na.omit(psmordt, cols=c(varweight, "Abundance"))
psm <- as.data.frame(psmdt)

# Occurrences, uk, abundance
gf1 <- as.factor(levels(psm[[taxopt]]))
ngen <- nlevels(gf1)
psm$Occurrences <- psm$Abundance
psm$Occurrences[psm$Occurrences > 0] <- 1
occurr <- numeric(ngen)
taxsum <- numeric(ngen)
x_Taxon <- character(ngen)
y_wa <- numeric(ngen)
i <- NULL
for (i in 1:ngen) {
  psm_s <- subset(psm, psm[[taxopt]] == gf1[i])
  y_wa[i] = weighted.mean(psm_s[[varweight]], psm_s$Abundance, na.rm = FALSE)
  x_Taxon[i] <- as.character(gf1[i])
  occurr[i] <- sum(psm_s$Occurrences)
  taxsum[i] <- sum(psm_s$Abundance)
  i <- i+1
 }
df1 <- data.frame(x_Taxon, occurr, taxsum, y_wa, stringsAsFactors=FALSE)
names(df1) <- c(taxopt, "Occurrences", "TotAbund", "uk")
psmm <- merge(psm, df1, by=taxopt)

# tk
psmm$fac <- (psmm[[varweight]] - psmm$uk)^2
x_Taxon <- character(ngen)
y_tk <- numeric(ngen)
i <- NULL
for (i in 1:ngen) {
  psmm_s <- subset(psmm, psmm[[taxopt]] == gf1[i])
  y_tk[i] = sqrt(weighted.mean(psmm_s$fac, psmm_s$Abundance, na.rm = FALSE))
  x_Taxon[i] <- as.character(gf1[i])
  i <- i+1
 }
df2 <- data.frame(x_Taxon, y_tk, stringsAsFactors=FALSE)
names(df2) <- c(taxopt, "tk")

# merge
optim_ps <- Reduce(function(x,y) merge(x = x, y = y, by = taxopt), list(df1, df2))
if (ranktax == TRUE) {
        taxdfm = as(tax_table(psg), "matrix")
        taxdf = as.data.frame(taxdfm)
        optim_ps_rt <- merge(optim_ps, taxdf, by = taxopt)
        return(optim_ps_rt)
        } else {
        return(optim_ps)
        }
}

### References
```


ter Braak, C. J. F., and van Dame, H. (1989). Inferring pH from diatoms: a comparison of old and new calibration methods. Hydrobiologia 178, 209–223. doi:10.1007/BF00006028.

Salmaso, N. (2003). Life strategies, dominance patterns and mechanisms promoting species coexistence in phytoplankton communities along complex environmental gradients. Hydrobiologia, 13–36. doi:10.1023/B:HYDR.0000004267.64870.85.
