# Supplementary material for: Effects of Habitat Partitioning on the Distribution of Bacterioplankton in Deep Lakes
Source: Front Microbiol. 2019 Oct 4;10:2257. doi: 10.3389/fmicb.2019.02257 (PMC6788347; doi:10.3389/fmicb.2019.02257)
Supplement: Supplementary file 3 [file Data_Sheet_3.PDF]

Supplementary Table 1 Differential distribution of genera along the vertical oxygen gradient. Species recorded with higher abundances (DESEQ2, *P*< 0.05) in (A) the epilimnetic and (B) the hypolimnetic oxygenated layers, and (C) the hypoxic and anoxic monimolimnia. Cumulative relative abundances (% on the respective totals) are reported under each lake and layer, and are highlighted as: light blue, 1% ≤ reads < 5%; orange, reads ≥ 5%. For each layer in (A), (B) and (C), families with abundances significantly different from the other layers were reported in bold (*P*<0.01) and italic (*P*<0.05). u_k and t_k are the species optima and species tolerances of oxygen concentrations (mg l^{-1}), respectively. Gar, Garda; Com, Como; Ise, Iseo; Lug, Lugano, Idr, Idro. epi_oxy, oxygenated upper layers (0-20 m); hyp_oxy, oxygenated hypolimnion; hyp_hypox, hypoxic/anoxic hypolimnion. Taxa names are based on the SILVA 132 taxonomy.

(A)

Phylum	Class	Order	Family	Genus	Gar	Com	Ise	Lug	Idr	epi_ oxy	hyp_ oxy	hyp_ hypox	u_k	t_k
Proteobacteria	Alphaproteobacteria	Acetobacterales	Acetobacteraceae	Roseomonas	2.99	0.15	0.90	0.37	0.92	4.94	0.36	0.02	10.99	1.43
	Alphaproteobacteria	Rhodobacterales	**Rhodobacteraceae**	Pseudorhodobacter	0.15	0.20	0	0.35	0.04	0.74	0	0	11.93	1.60
	Alphaproteobacteria	Sphingomonadales	Sphingomonadaceae	Sandarakinorhabdus	1.25	0.38	0	6.70	0.62	8.88	0.08	0	10.87	1.77
	Gammaproteobacteria	Betaproteobacteriales	Burkholderiaceae	Lautropia	0.36	0	2.13	0	0.46	2.91	0.04	0	10.12	1.05
	Gammaproteobacteria	Betaproteobacteriales	Burkholderiaceae	Polaromonas	1.14	2.17	0.41	1.53	0.47	5.32	0.39	0	11.35	1.61
Actinobacteria	Actinobacteria	Micrococcales	**Microbacteriaceae**	Candidatus_Aquiluna	0.49	0	0.32	1.45	0.80	3.00	0.07	0	11.03	1.19
	Actinobacteria	Micrococcales	**Microbacteriaceae**	Candidatus_Planktoluna	0	0.70	0	0.01	0.12	0.83	0	0	11.23	0.66
Bacteroidetes	Bacteroidia	Flavobacteriales	**Flavobacteriaceae**	Flavobacterium	21.6	9.31	1.07	30.9	7.88	64.9	5.42	0.43	11.19	1.48
Cyanobacteria	Oxyphotobacteria	Nostocales	**Nostocaceae**	Aphanizomenon (Dolichospermum)	0.22	0	41.1	0	0.75	41.6	0.37	0.03	8.65	0.49
Spirochaetes	Leptospirae	Leptospirales	**Leptospiraceae**	Leptospira	0.03	0.28	0.21	0	0.09	0.61	0	0	10.68	0.86

(B)

Phylum	Class	Order	Family	Genus	Gar	Com	Ise	Lug	Idr	epi_ oxy	hyp_ oxy	hyp_ hypox	uk	tk
Proteobacteria	Alphaproteobacteria	Rhizobiales	Beijerinckiaceae	Methylocystis	0.09	1.05	0	0	0	0	1.14	0	9.72	0.95
	Alphaproteobacteria	SAR11_clade	**Clade_I**	Clade_Ia	1.50	0.49	0.07	0	0	0	2.06	0	9.27	1.31
	Gammaproteobacteria	Alteromonadales	**Alteromonadaceae**	Rheinheimera	1.13	0.58	0.07	0.12	0	0.03	1.79	0.08	8.34	2.28
	Gammaproteobacteria	Betaproteobacteriales	*Nitrosomonadaceae*	MND1	5.46	2.06	0.36	0.26	0.12	0.18	7.99	0.09	8.55	1.70
Acidobacteria	Acidobacteriia	Solibacterales	*Solibacteraceae (Subgroup_3)*	Bryobacter	0.64	0.19	0	0.03	0	0	0.87	0	9.07	1.33
	Acidobacteriia	Solibacterales	*Solibacteraceae (Subgroup_3)*	Candidatus_Solibacter	0.17	0.27	0.33	0.15	0.02	0.07	0.86	0	8.34	1.92
Actinobacteria	Acidimicrobiia	Microtrichales	**Iamiaceae**	Iamia	0.52	0.90	0.06	0.09	0	0	1.57	0	8.73	1.65
Armatimonadetes	Chthonomonadetes	Chthonomonadales	**Chthonomonadaceae**	Chthonomonas	0.05	0.66	0.16	0.07	0	0	0.93	0	8.91	1.68
Bacteroidetes	Bacteroidia	Chitinophagales	Saprospiraceae	Haliscomenobacter	1.23	0	0	0	0	0	1.23	0	7.07	1.10
Planctomycetes	Planctomycetacia	Gemmatales	**Gemmataceae**	Gemmata	0.99	1.18	0.58	0.17	0	0.02	2.89	0	8.86	1.63

(C)

Phylum	Class	Order	Family	Genus	Gar	Com	Iseo	Lug	Idro	epi_ oxy	hyp_ oxy	hyp_ hypox	uk	tk

Proteobacteria	Deltaproteobacteria	Desulfobacterales	**Desulfobacteraceae**	Desulfatirhabdium	0	0	1.04	0.94	0.52	0	0	2.50	0.08	0.16
	Deltaproteobacteria	Desulfobacterales	**Desulfobacteraceae**	Desulfobacula	0	0	0.29	0.55	0	0	0	0.84	0.05	0.04
	Deltaproteobacteria	Desulfobacterales	**Desulfobacteraceae**	Sva0081_sediment _group	0.01	0	0.01	0.09	0.66	0	0.01	0.76	0.35	0.79
	Deltaproteobacteria	Desulfobacterales	**Desulfobulbaceae**	Desulfurivibrio	0	0	0	0	5.32	0	0	5.32	0.58	0.62
	Deltaproteobacteria	Syntrophobacterales	**Syntrophaceae**	Desulfobacca	0	0	0	11.16	2.42	0	0.02	13.55	0.12	0.37
	Deltaproteobacteria	Syntrophobacterales	**Syntrophaceae**	Desulfomonile	0	0	0	0	15.40	0.01	0	15.39	0.22	0.30
	Deltaproteobacteria	Syntrophobacterales	**Syntrophaceae**	Syntrophus	0	0	0.16	3.61	0.86	0	0	4.63	0.12	0.25
	Gammaproteobacteria	Betaproteobacteriales	Gallionellaceae	Gallionella	0	0	0	5.04	0	0	0	5.04	0.05	0.04
	Gammaproteobacteria	Betaproteobacteriales	**Methylophilaceae**	Methylotenera	0.33	2.76	3.76	23.75	14.16	0.03	4.35	40.38	1.42	2.70
	Gammaproteobacteria	Betaproteobacteriales	**Rhodocyclaceae**	Dechloromonas	0	0	0	0.59	0	0	0	0.59	0.07	0.08
	Gammaproteobacteria	Betaproteobacteriales	**Rhodocyclaceae**	Denitratisoma	0	0	8.37	1.54	0.05	0	0.13	9.83	0.26	0.70
	Gammaproteobacteria	Betaproteobacteriales	**Rhodocyclaceae**	Sterolibacterium	0	0	1.23	1.20	0	0	0.05	2.38	0.37	0.91
	Gammaproteobacteria	Betaproteobacteriales	**Rhodocyclaceae**	Sulfuritalea	0.01	0	1.56	0.19	0.42	0	0.01	2.17	0.47	1.03
	Gammaproteobacteria	Methylococcales	**Methylomonaceae**	Crenothrix	0.88	1.73	0.70	26.95	8.49	0	2.83	35.93	1.16	2.45
	Gammaproteobacteria	Methylococcales	**Methylomonaceae**	Methyloglobulus	0	0	0.29	0.55	0.14	0	0.05	0.94	0.67	1.33
	Gammaproteobacteria	Methylococcales	**Methylomonaceae**	pLW-20	0	0	1.17	20.78	1.60	0.06	0.73	22.76	0.50	1.26
Epsilonbacteraeota	Campylobacteria	Campylobacterales	**Thiovulaceae**	Sulfuricurvum	0	0	0.03	0.06	69.12	0	0	69.21	0.26	0.17
	Campylobacteria	Campylobacterales	**Thiovulaceae**	Sulfurimonas	0	0	0.20	0	9.67	0	0	9.87	0.30	0.33
Bacteroidetes	Bacteroidia	Bacteroidales	**Paludibacteraceae**	Paludibacter	0	0	2.64	0.12	0.48	0.04	0	3.20	0.12	0.62
	Bacteroidia	Bacteroidales	**Prolixibacteraceae**	BSV13	0	0	5.23	2.99	1.25	0	0	9.48	0.24	0.43
Chlamydiae	Chlamydiae	Chlamydiales	*Parachlamydiaceae*	Candidatus Protochlamydia	0	0.05	0.97	0.01	0	0.01	0.05	0.97	1.02	3.02
Chloroflexi	Anaerolineae	Anaerolineales	Anaerolineaceae	GWD2-49-16	0	0	0	1.81	0.01	0	0	1.83	0.07	0.16
	Anaerolineae	Anaerolineales	Anaerolineaceae	Longilinea	0	0	0	0.58	1.69	0	0	2.27	0.21	0.31
	Anaerolineae	Anaerolineales	Anaerolineaceae	Pelolinea	0	0	0	0.69	0.04	0	0	0.73	0.06	0.08
Firmicutes	Clostridia	Clostridiales	**Family_XIII**	Anaerovorax	0	0	0.59	0.03	0.03	0	0	0.64	0.05	0.02
Kiritimatiellaeota	Kiritimatiellae	Kiritimatiellales	**Kiritimatiellaceae**	MSBL3	0	0	0.35	1.78	1.60	0	0	3.73	0.16	0.30
Planctomycetes	Planctomycetacia	Pirellulales	Pirellulaceae	Candidatus Anammoximicrobium	0	0	0	1.23	0.99	0	0	2.22	0.23	0.43
Rokubacteria	NC10	Methylomirabilales	**Methylomirabilaceae**	Candidatus Methylomirabilis	0	0	12.12	5.92	0	0	0.11	17.93	0.19	0.49
	NC10	Methylomirabilales	**Methylomirabilaceae**	Sh765B-TzT-35	0	0	0	0.87	0.39	0	0	1.26	0.11	0.11
Verrucomicrobia	Verrucomicrobiae	Opitutales	Opitutaceae	Opitutus? (Cephaloticoccus)	0	0	1.04	0.45	0.55	0	0	2.04	0.23	0.40
	Verrucomicrobiae	Pedosphaerales	Pedosphaeraceae	ADurb.Bin063-1	0	0	0.08	0.04	0.20	0	0	0.32	0.73	0.74
