# Supplementary figures and images for: Effects of Habitat Partitioning on the Distribution of Bacterioplankton in Deep Lakes
Source: Front Microbiol. 2019 Oct 4;10:2257. doi: 10.3389/fmicb.2019.02257 (PMC6788347; doi:10.3389/fmicb.2019.02257)

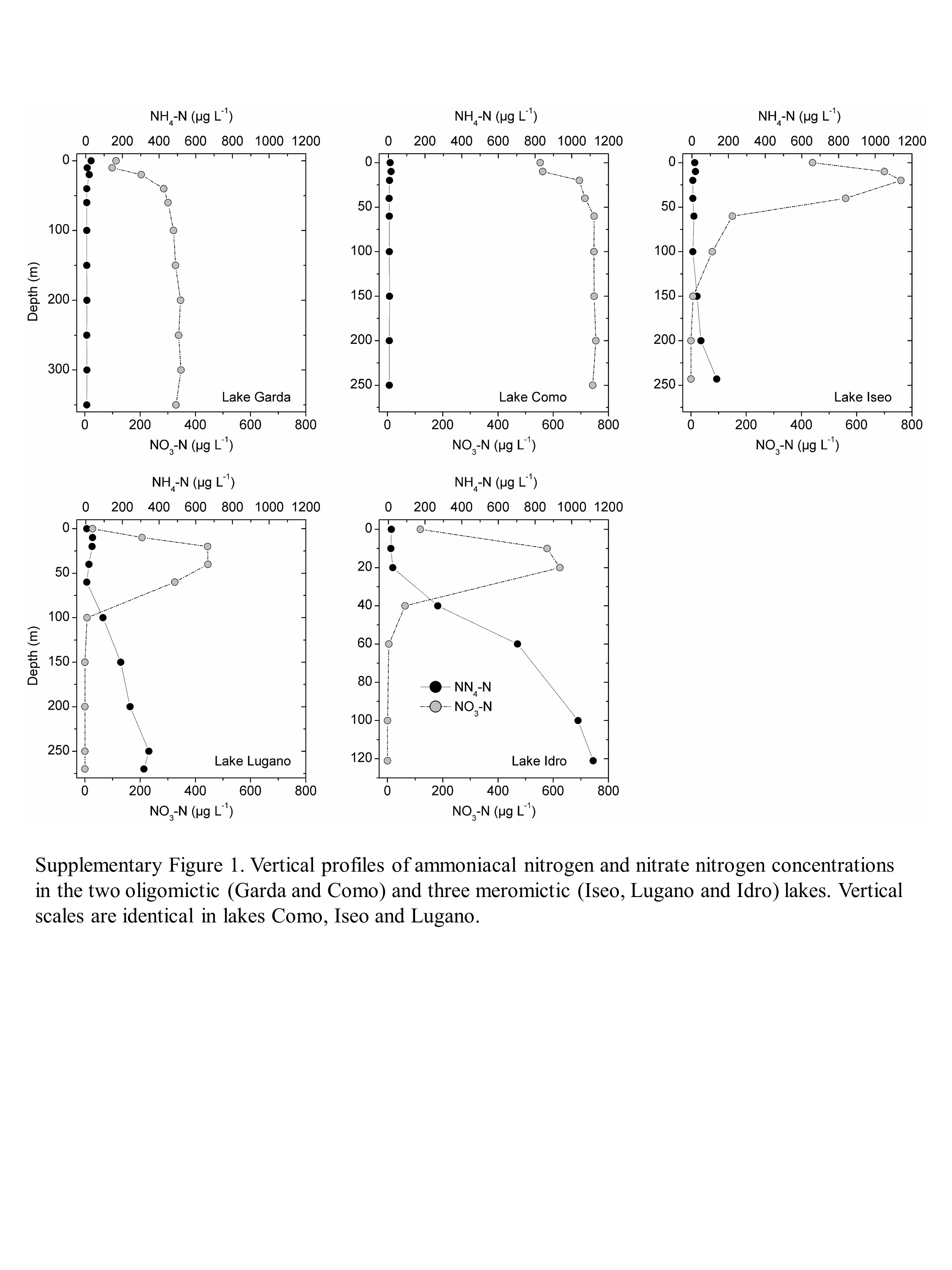

Supplement: Supplementary file 4 [file Image_1.TIF]

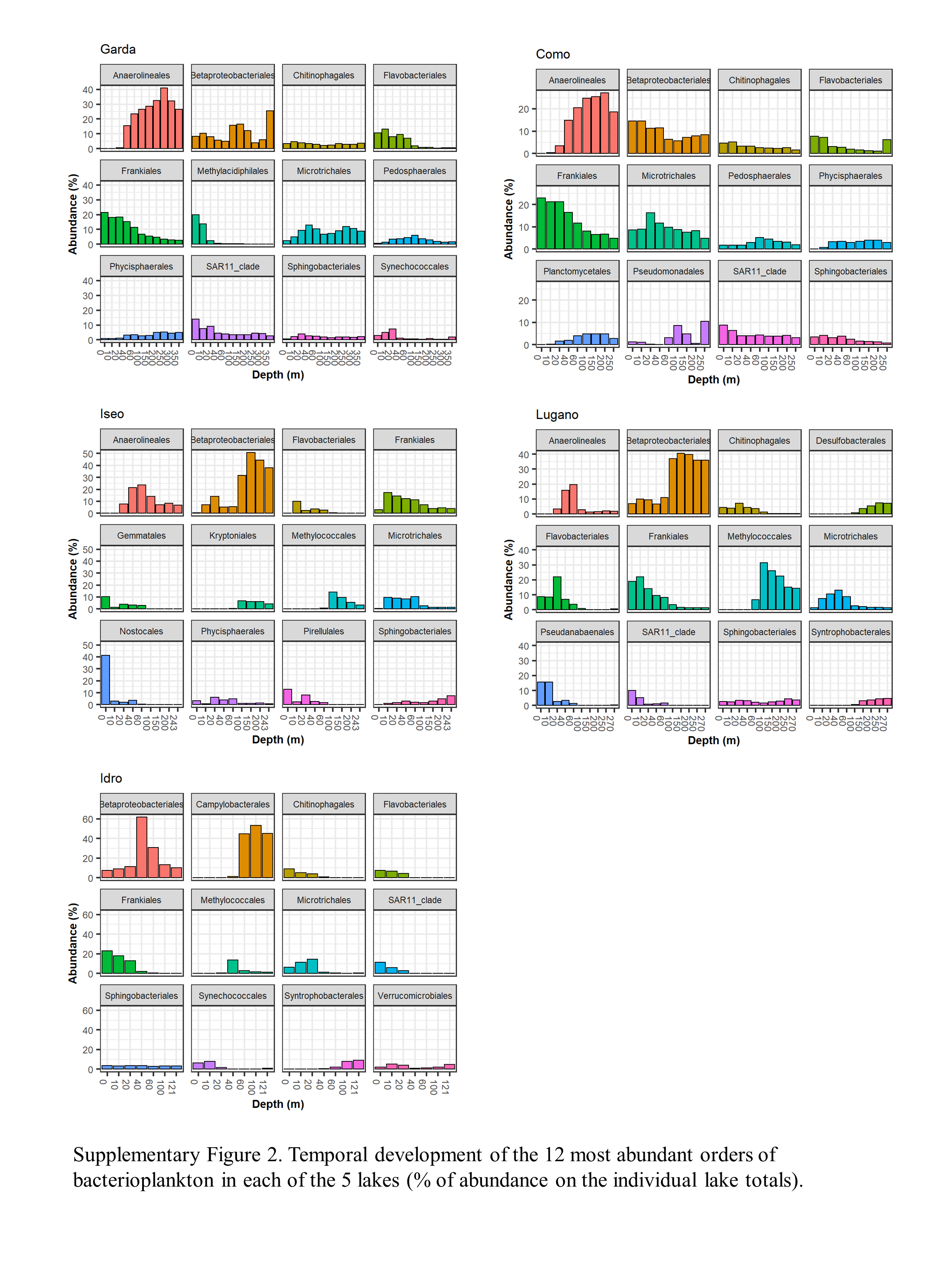

Supplement: Supplementary file 5 [file Image_2.TIF]

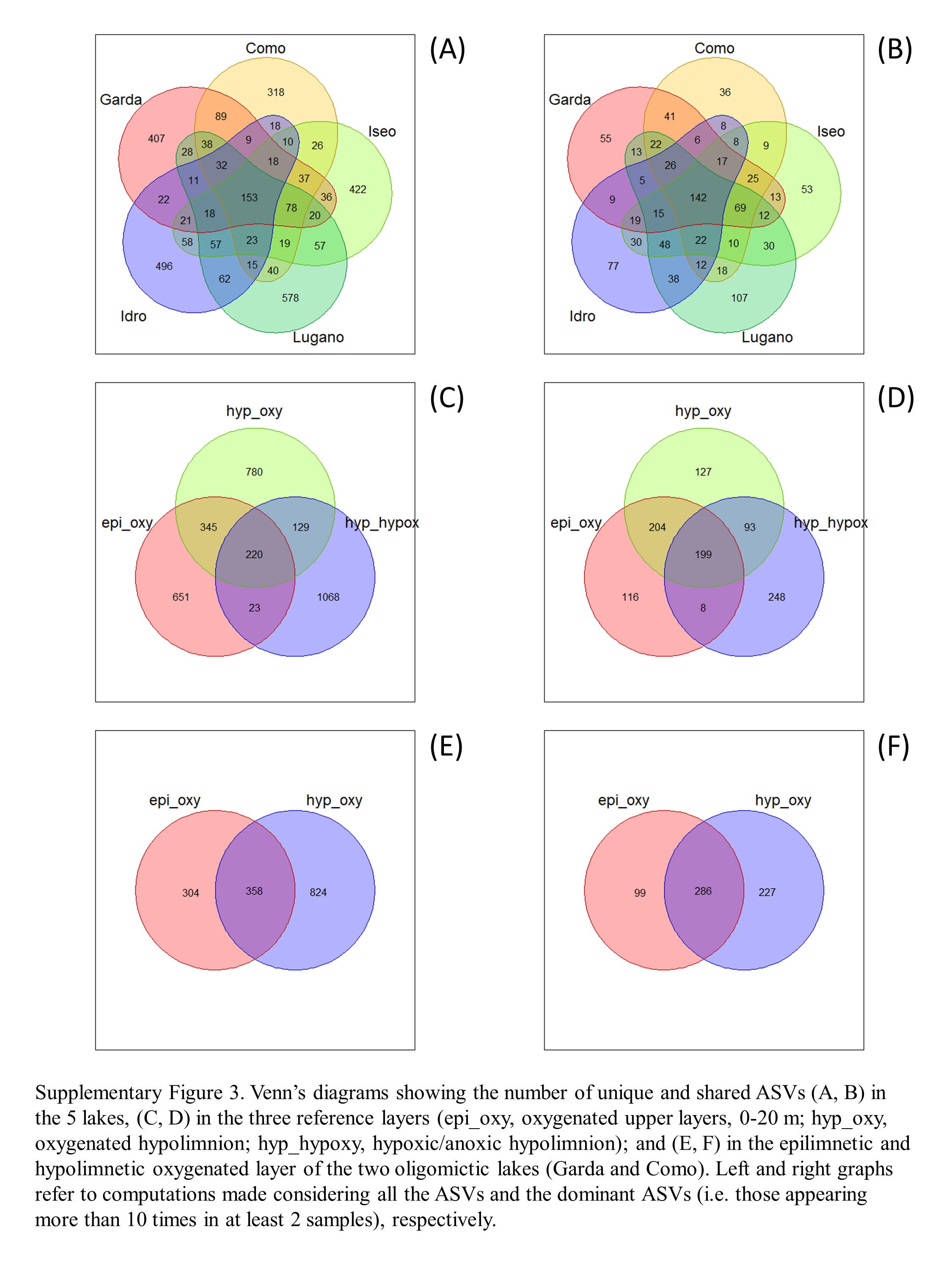

Supplement: Supplementary file 6 [file Image_3.TIF]

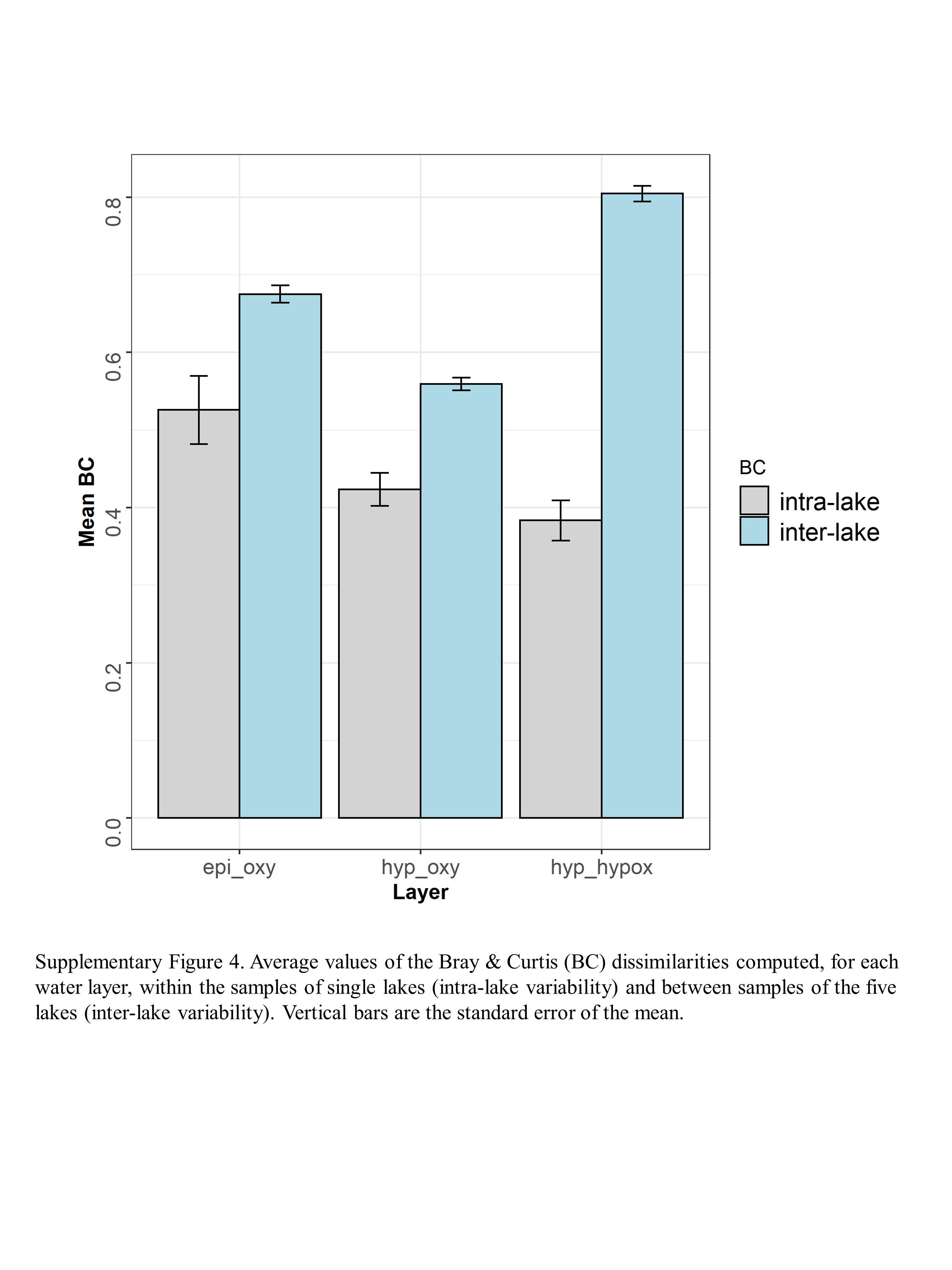

Supplement: Supplementary file 7 [file Image_4.TIF]
